# Biomechanical simulations of hindlimb function in *Alligator* provide insights into postural shifts and body size evolution

**DOI:** 10.1126/sciadv.adx3811

**Published:** 2025-10-22

**Authors:** Masaya Iijima, Richard W. Blob, John R. Hutchinson

**Affiliations:** ^1^ Key Laboratory of Vertebrate Evolution and Human Origins, Institute of Vertebrate Paleontology and Paleoanthropology, Chinese Academy of Sciences, Beijing 100044, China.; ^2^ Structure and Motion Laboratory, Department of Comparative Biomedical Sciences, The Royal Veterinary College, Hawkshead Lane, North Mymms AL9 7TA, UK.; ^3^ Department of Natural Environmental Studies, Graduate School of Frontier Sciences, The University of Tokyo, Kashiwa 277-0882, Japan.; ^4^ Department of Biological Sciences, Clemson University, Clemson, SC 29634, USA.

## Abstract

The shift from sprawling to erect limb postures in archosaurs during the Triassic represents a major evolutionary transformation in vertebrates. One unresolved question regarding the limb posture transition is its association with body size evolution. If adopting more erect limb postures reduces mass-specific muscle forces and bone stresses, it would enable the evolution of larger body sizes. We tested this prediction using computational modeling and simulation of hindlimb muscle activations and femoral stresses across limb postures and body sizes in juvenile to adult American alligators and a scaled model of the extinct giant alligatoroid *Deinosuchus riograndensis*. We showed that larger alligators and *D. riograndensis* encounter challenges in generating sufficient muscle forces to support their bodies and maintain bone stresses, whereas adopting more erect hindlimb postures helps mitigate bone stresses among individuals of similar sizes. These results show how the shift from sprawling to erect limb posture relaxed biomechanical constraints, potentially facilitating the evolution of larger body sizes in terrestrial tetrapods.

## Teaser

Locomotor simulations in alligators reveal that transitions to erect limb postures facilitate the evolution of larger body sizes.

## INTRODUCTION

The shift from sprawling postures, with limbs projected sideways, to erect postures, with limbs held beneath the body, represents a major evolutionary transformation that occurred in two major tetrapod lineages, archosaurs and synapsids. During the Permian and Triassic, both groups independently underwent transitions to more erect limb postures, as evidenced by changes in appendicular musculoskeletal architectures (*1*–*10*), joint mobilities (*11*–*14*), muscle leverages and force-generating capacities (*15*, *16*), bone stresses (*17*, *18*), and footprints (*19*–*21*). The evolution of erect limb postures and parasagittally moving limbs allowed breathing while running, thereby enhancing aerobic locomotor capacities (*22*). As improved locomotor stamina demands higher resting metabolic rates (*23*), the adoption of erect limb postures may have also contributed to the evolution of endothermy in these lineages (*22*, *24*, *25*). Furthermore, erect limb postures facilitated the evolution of bipedality in archosaurs (*26*–*28*), which paved the way for the acquisition of powered flight in paravian dinosaurs (*29*–*31*).

Another possible, although less discussed, consequence of the transition to erect limb postures is the evolution of larger body sizes, which is enabled by the release from size-related biomechanical constraints. In mammals and birds with parasagittal limbs, smaller species adopt more crouched postures, while larger species adopt more upright postures (*32*–*36*). By aligning their limbs closely to the ground reaction force, larger mammals and birds can reduce muscle forces and mitigate soft tissue and bone stresses (*32*–*34*). The posture-related mechanism to mitigate muscle forces and tissue loading may be relevant not only to the crouched-to-upright posture gradient in animals with parasagittal limbs but also to the sprawling-to-erect posture gradient in animals with nonparasagittal limbs. If larger tetrapods with nonparasagittal limbs tend to use more erect limb postures, then it may suggest that transitions from sprawling to erect postures are a general mechanism that enables the evolution of larger body sizes.

Among animals with nonparasagittal limbs, American alligators demonstrate ontogenetic changes in limb postures. Although alligators are capable of using a range of limb postures on land, from sprawling to high-walk (*37*, *38*), larger individuals tended to favor more erect postures during steady-speed walking [(*39*, *40*); yet see (*41*) for an example with no size-related changes in limb posture in varanid lizards]. However, the effects of size and posture on the functions of supportive tissues imposing ultimate constraints on locomotion, such as muscle activations and bone loading, remain unclear. Previous electromyography (EMG) and bone strain recordings in juvenile alligators yielded mixed results: Some studies supported the expectation that muscle excitation and bone loading increase with body size when similar postures are adopted and that both decrease within individual animals when more erect postures were used (*42*, *43*), whereas other studies showed contradictory posture-related trends (*44*, *45*). Yet, these invasive experiments posed several challenges, including potential alterations in locomotor behavior caused by surgical interventions, limited coverage of target muscles, and the absence of data from larger individuals (*42*, *43*).

**Fig. 1.**
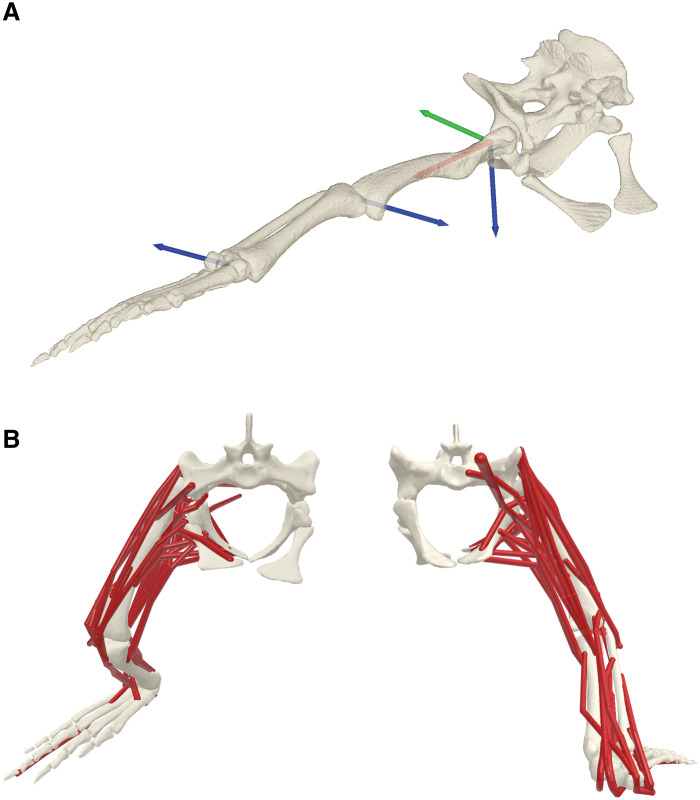
Hindlimb musculoskeletal model of the American alligator. (**A**) Hindlimb joint coordinate systems (JCSs) in a neutral pose (reference; all angles, 0°). The rotation order of the axes is *z* (blue), *y* (green), and *x* (red), with the right-hand rule applied to define the rotation signs. The hip joint has three rotational degrees of freedom (extension-flexion, abduction-adduction, and femoral long-axis rotation), while the knee and ankle joints each have one rotational degree of freedom (extension-flexion). (**B**) Thirty-six hindlimb musculotendon actuators in anterior (left) and posterior (right) views.

To better understand the biomechanical significance of limb posture transition and its association with body size evolution, here, we use computational musculoskeletal modeling (Fig. 1) to estimate muscle activations and femoral bone loading in the hindlimbs of juvenile to adult alligators as they walk with different limb postures. Additionally, hindlimb biomechanics of the extinct giant alligatoroid *Deinosuchus riograndensis* (3.7 tonnes; fig. S1) were estimated using a scaled model based on extant alligators. Leveraging computational modeling and subject-specific experimental data, our analyses cover a body size range spanning four orders of magnitude, targeting most of hindlimb muscles and grounded in naturalistic locomotor behavior. We find that larger alligators and *Deinosuchus* experience greater limitations in force-generating capacities and increased femoral stresses, which would be even more pronounced without the adoption of more erect postures by such larger individuals. Additionally, femoral stresses decrease with more adducted (erect) hip joint postures among individuals of similar sizes. These findings support the prediction that the shift from sprawling to erect posture alleviated mechanical constraints and facilitated the evolution of larger body sizes in archosaurs and potentially other lineages such as synapsids.

## RESULTS

### Hindlimb postures and joint moments

During stance phase, small juvenile alligators (0.23 to 0.25 kg) typically adopted more crouched limb postures with a less adducted hip and a more flexed knee, compared to larger juvenile and adult alligators (>1.0 kg) (fig. S3A). Consequently, normalized hip abduction moments were greater in small juveniles than larger individuals, whereas normalized knee flexion moments did not significantly differ between these size groups (fig. S3B).

### Hindlimb muscle activations

**Fig. 2.**
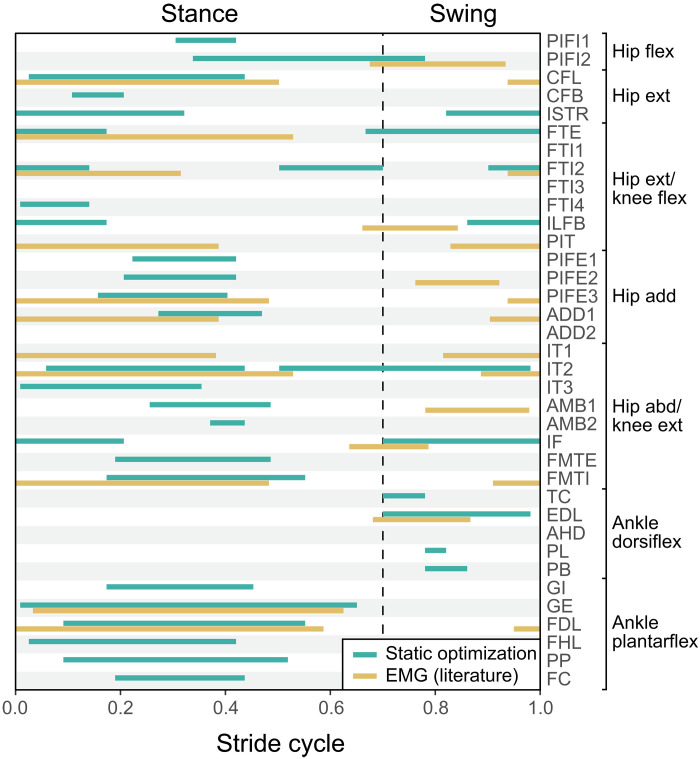
Comparison of estimated activation patterns and recorded EMG excitation patterns in the hindlimb muscles of American alligators. Activation patterns of 36 hindlimb muscles were estimated through static optimization on the basis of a representative trial from a juvenile alligator (al05f90). EMG excitation patterns for available muscles were sourced from previous studies (*45*–*47*). The stance-to-stride ratio was adjusted to 0.7 for comparisons. Full muscle names are provided in table S2. Joint motion abbreviations: add, adduction; abd, abduction; dorsiflex, dorsiflexion; ext, extension; flex, flexion; plantarflex, plantarflexion.

The activation timings of hindlimb muscles estimated through static optimization–based inverse simulations were largely consistent with previous in vivo recording of muscle excitations via EMG (Fig. 2) (*45*–*47*). However, the degree of overlap between the simulated and EMG patterns varied, with iliofibularis and ambiens 1 showing no overlap: Iliofibularis was active during the stance-to-swing transition in simulation, contrary to its excitation during the swing-to-stance transition in EMG, while ambiens 1 was active during midstance in simulation despite being excited during midswing in EMG (Fig. 2). Muscles were functionally categorized according to their predicted functions on the basis of their paths and attachment sites, as well as simulated activation patterns (Fig. 2). Hip extensors (caudofemoralis longus and brevis and ischiotrochantericus), hip adductors (puboischiofemoralis externus and adductor femoris), knee extensors (e.g., iliotibialis and femorotibialis), and ankle plantarflexors (e.g., gastrocnemius and flexor digitorum longus), which generate propulsive and antigravity forces, were primarily active during stance. In contrast, knee flexors (e.g., flexor tibialis externus and internus) and ankle dorsiflexors (e.g., tibialis cranialis and extensor digitorum longus) were mainly active during swing and the swing-to-stance transition (Fig. 2). Note that puboischiofemoralis internus 1 and 2 were categorized as hip flexors, despite showing stance-phase activity, possibly acting as hip stabilizers.

**Fig. 3.**
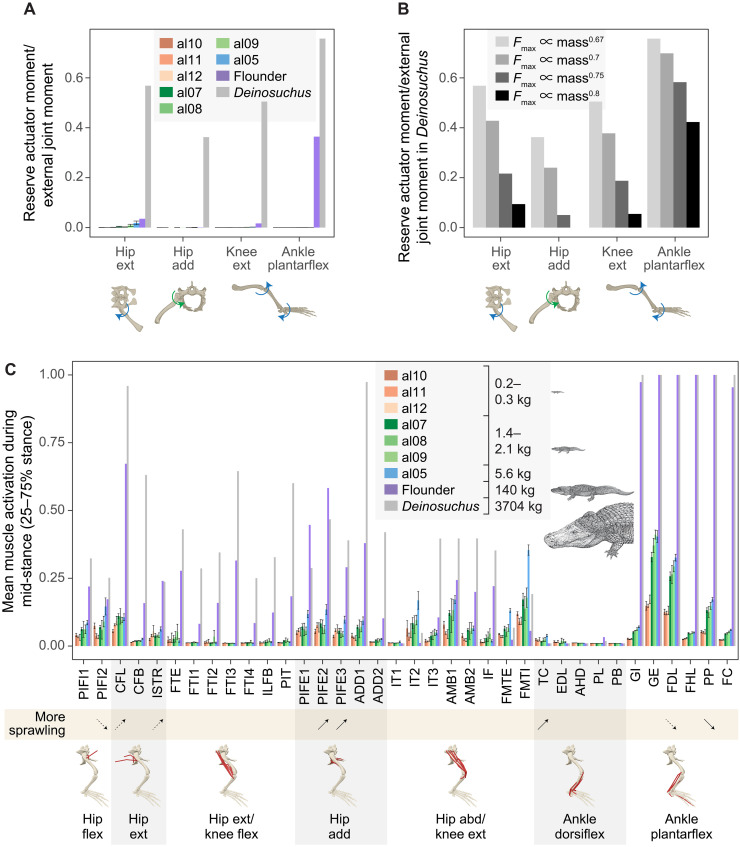
Hindlimb muscle force-generating capacities in American alligators and *D. riograndensis* across body sizes. (**A**) Ratios of reserve actuator moment to corresponding external joint moment at the instant of peak joint moment for major joint motions, including hip extension and adduction, knee extension, and ankle plantarflexion in eight juvenile to adult American alligators and *D. riograndensis* (TMM43632-1). (**B**) Changes in the ratios of reserve actuator moment to external joint moment with four different *F*_max_ scaling factors (*F*_max_ scaled as the 0.67, 0.7, 0.75, and 0.8 power of body mass from the base model) for *D. riograndensis* (TMM43632-1). (**C**) Mean activations of 36 hindlimb muscles during midstance (25 to 75% stance) in eight juvenile to adult American alligators and *D. riograndensis* (TMM43632-1). The bar plot and error bar represent the means ± SEM for each individual. Muscle activations are scaled from 0 to 1. Upturned and downturned arrows in the beige band indicate increased and decreased activations, respectively, with more sprawling (abducted) hip postures in three small juveniles (al10, al11, and al12; body masses, 0.23 to 0.25 kg). Solid arrows represent significant (*P* < 0.05) correlations, whereas dashed arrows represent marginal (0.05 ≤ *P* < 0.1) correlations between muscle activations and hip postures, as determined by linear regressions (table S3). Full muscle names are provided in table S2. Joint motion abbreviations: add, adduction; abd, abduction; dorsiflex, dorsiflexion; ext, extension; flex, flexion; plantarflex, plantarflexion. Illustrations of *Alligator* and *Deinosuchus* are by Takashi Oda; used with permission.

Among reserve actuators, which are torques that compensate for insufficient muscle forces and unaccounted passive tissue forces (*48*), the hip extensor, hip adductor, and knee extensor reserve actuator moments in alligators of all sizes remained below 5% of their respective joint moments from external forces at the instant of the peak joint moment (Fig. 3A). Note that the femur axial rotation reserve actuator, which counteracts relatively small external joint moments (fig. S3B), was not assessed here. In contrast, the ankle plantarflexor reserve actuator moment in the adult alligator (140 kg) reached 36% of the corresponding external joint moment (Fig. 3A). Sensitivity analysis with the initial center of pressure (CoP) positioned closer to the ankle yielded a similar result: The hip extensor, hip adductor, and knee extensor reserve actuator moments remained below 5% of their respective external joint moments, while the ankle plantarflexor reserve actuator moment reached 33% of the corresponding external joint moment in the adult alligator (fig. S4A). In contrast to the trend observed in alligators, all those reserve actuator moments in *Deinosuchus* exceeded 20% of their corresponding external joint moments under isometric *F*_max_ scaling (∝ mass^0.67^) from the base alligator model, regardless of the initial CoP position (Fig. 3A and fig. S4A). To align the ratios of the reserve actuator moment to corresponding external joint moment in *Deinosuchus* to those observed in the adult alligator, *F*_max_ needed be scaled as the 0.8 power of body mass or higher in *Deinosuchus* from the base alligator model, when the default initial CoP is used (i.e., strong allometry, contradicting the isometric scaling present in extant Crocodylia) (Fig. 3B) (*49*, *50*).

The mean midstance activations (scaled from 0 to 1) of the hip flexors, extensors, and adductors, knee extensors, and ankle plantarflexors—muscles primarily responsible for generating propulsive and antigravity forces, with the exception of the hip flexors—were generally higher than those of the knee flexors and ankle dorsiflexors in each individual (Fig. 3C). When comparing mean muscle activations across different sizes, larger individuals exhibited higher activations for all muscles (Fig. 3C). It is noteworthy that activations of most ankle plantarflexors approached 1.0 in the adult alligator (140 kg) and *Deinosuchus* (3.7 tonnes), whereas the antagonistic ankle dorsiflexors maintained low activity levels. Peak stance-phase activations of muscles generating propulsive and antigravity forces were also generally higher than other muscles in each individual, and these activations increased with size, approaching 1.0 in the adult alligator and *Deinosuchus* (fig. S4B).

Activation levels were correlated with hip adduction angles in several muscles in small juveniles of similar sizes (0.23 to 0.25 kg). As they adopted more sprawling (abducted) hip postures, the activations of the hip extensors caudofemoralis longus and ischiotrochantericus, hip adductors puboischiofemoralis externus 2 and 3, and ankle dorsiflexor tibialis cranialis increased significantly (*P* < 0.05) or marginally (0.05 ≤ *P* < 0.1) (Fig. 3C, fig. S5, and table S3). Meanwhile, the activations of the hip flexor puboischiofemoralis internus 2 and the ankle plantarflexors flexor digitorum longus and pronator profundus decreased significantly or marginally (Fig. 3C and table S3).

### Femoral stresses

**Fig. 4.**
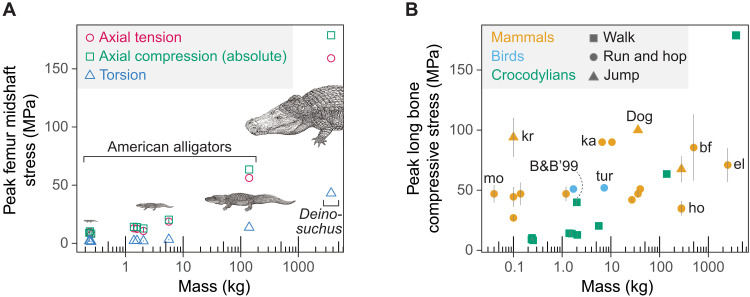
Limb bone stresses in crocodylians (American alligators and *D. riograndensis*), mammals, and birds across body sizes. (**A**) Peak femoral midshaft stresses (megapascals), including axial tension, axial compression (absolute values), and torsion in eight juvenile to adult American alligators and *D. riograndensis* (TMM43632-1). (**B**) Peak midshaft compressive stresses (megapascals) in the limb bones of mammals, birds, and crocodylians (American alligators and *D. riograndensis*) (*44*, *68*, *123*–*133*). B&B’99 refers to bone stresses derived from in vivo bone strains of juvenile American alligators in (*44*). Error bars represent the SEM. Animal abbreviations: bf, buffalo; el, elephant; ho, horse; ka, red kangaroo; kr, kangaroo rat; mo, mouse; tur, turkey. Illustrations of *Alligator* and *Deinosuchus* are by Takashi Oda; used with permission.

Femoral midshaft stresses increased with body size (Fig. 4A and table S4). Mean peak midshaft axial tensile stresses in the femur increased with body size, ranging from 7.7 to 10.0 MPa in small juveniles (0.23 to 0.25 kg), 10.7 to 18.7 MPa in larger juveniles (1.4 to 5.6 kg), 56.2 MPa in the adult (140 kg), to 159 MPa in *Deinosuchus* (3.7 tonnes). When the bone stresses were calculated with the initial CoP shifted closer to the ankle, the mean peak midshaft axial tensile stresses decreased by an average of 9.9% (from 7.3 to 11.4 MPa in small juveniles, 10.4 to 18.6 MPa in larger juveniles, 43.7 MPa in the adult, to 96.1 MPa in *Deinosuchus*) (fig. S6A and table S4). Peak midshaft axial compressive stresses were slightly greater in magnitude than peak midshaft axial tensile stresses and followed a similar size-related trend, with an average of 11.6% reduction in the stress magnitudes in the CoP sensitivity analysis (Fig. 4A, fig. S6A, and table S4). The neutral axis of bending was diagonally oriented relative to the mediolateral axis of the midshaft cross-section, with tension dominating on the ventromedial cortex and compression dominating the dorsolateral cortex during midstance (fig. S7A). Peak torsional stresses (in absolute values) were much smaller than peak midshaft axial tensile and compressive stresses but also increased with body mass, ranging from 1.4 to 3.3 MPa in juveniles (0.2 to 5.6 kg), 13.7 MPa in the adult (140 kg), to 43.1 MPa in *Deinosuchus* (3.7 tonnes) (Fig. 4A and table S4). Peak torsional stresses increased by an average of 22.6% in the CoP sensitivity analysis (fig. S6A and table S4). Femoral midshaft stresses in *Deinosuchus* showed minimal sensitivity to the *F*_max_ scaling. When *F*_max_ was scaled as mass^0.8^ from the base alligator model, the resulting femoral stresses were comparable to those calculated using the isometric *F*_max_ scaling (∝ mass^0.67^) with the default initial CoP (fig. S6B).

Comparisons of peak compressive stresses in the femur of alligators to those in the long bones of mammals and birds during strenuous activities show that limb bone stresses in juvenile and adult alligators were either smaller than or comparable to those in mammals and birds of similar sizes (Fig. 4B). In contrast, the femoral bone stress in *Deinosuchus* exceeds the bone stresses previously recorded in large mammals.

**Fig. 5.**
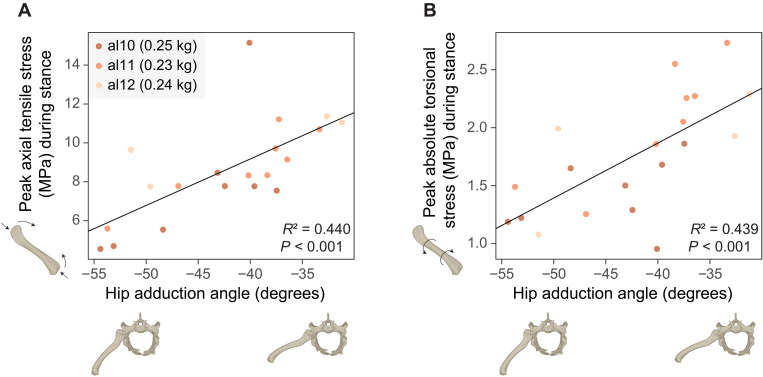
Posture-related changes in femoral stresses in juvenile American alligators. (**A**) Biplot of peak femoral axial tensile stress (megapascals) during stance and hip adduction angle (degrees), with the linear regression line for three small juveniles (al10, al11, and al12; body masses, 0.23 to 0.25 kg). (**B**) Biplot of peak femoral absolute torsional stress (megapascals) during stance and hip adduction angle (degrees), with the linear regression line for three small juveniles (al10, al11, and al12; body masses, 0.23 to 0.25 kg).

Femoral midshaft stresses were also dependent on limb posture. Peak midshaft axial tensile and compressive stresses, as well as peak torsional stresses, increased in magnitude with more sprawling (abducted) hip postures (Fig. 5 and table S5). However, the ratio of peak midshaft axial tensile to torsional stresses did not correlate with hip adduction angle (table S5).

## DISCUSSION

### Evaluating simulated hindlimb muscle activations and femoral stresses

The overall agreement between muscles’ predicted activations and measured EMG excitation timings (Fig. 2) supports our use of static optimization for estimating muscle functions, despite limitations such as the neglect of activation dynamics and tendon compliance, assumptions in optimization criteria, and its inaccuracies in predicting antagonist muscle cocontraction (*48*, *51*, *52*). Additionally, bone stresses computed from joint reaction forces and moments at the femoral midshaft closely aligned with in vivo bone loadings (fig. S7). The simulated orientations of the neutral axis of bending are consistent with those derived from bone strains (*44*), and the mean peak midshaft axial tensile stresses (10.7 to 18.7 MPa; table S4) estimated in larger juveniles weighing 1.4 to 5.6 kg are comparable to the 12.4 MPa derived from the peak midshaft axial tensile strain (1027 με) of the femur during stance in juvenile alligators weighing 1.7 to 2.3 kg (*44*).

### Size-related changes in hindlimb muscle activations and femoral stresses

Larger alligators experience greater limitations in hindlimb muscle force capacities, consistent with the scaling of limb bone proportions and muscle properties, as well as the size-related decline in terrestrial athleticism observed in extant crocodylians (*49*, *53*–*55*). Among the hindlimb muscles activated during stance, the ankle plantarflexors were the most highly recruited in each individual (with antagonistic dorsiflexors nearly silent) and reached their maximum capacities more quickly than other muscles as body size increased (Fig. 3C). The deficiency in plantarflexor muscle force capacities was particularly pronounced in the adult alligator (140 kg), with muscle moments falling 36% short of the required joint moments (33% short when the initial CoP shifted closer to the ankle) (Fig. 3A and fig. S4A). This might result from unaccounted passive structures in the ankle and challenges in modeling complex musculotendon architecture. For instance, the caudofemoralis longus in crocodylians inserts on the fourth trochanter, while its branched tendon extends distally and further splits into two: one inserts on the lateral femoral epicondyle and the other joins the gastrocnemius externus (*56*, *57*). This configuration enables the caudofemoralis longus to serve as both a hip extensor and potentially an ankle plantarflexor. Moreover, if the gastrocnemius externus is considered as a passive element with no length change, then the caudofemoralis longus and its branched tendons form a passive interlocking system that helps to maintain a more erect limb posture during stance (*58*). Meanwhile, for the adult alligator, the ankle plantarflexor fibulocalcaneus was activated at suboptimal normalized fiber lengths [*ĩ*_0_ below 0.7; (*59*)] during part of midstance, due to a more plantarflexed ankle compared to smaller alligators (fig. S3A), diminishing its force capacities. While unaccounted forces from passive structures and suboptimal fiber lengths may partially account for deficiencies in generating the required joint moments, the capacities of ankle plantarflexors to produce joint moments may still be smaller compared to muscles at other joints—a pattern also observed in extant tetrapods during walking, running, and sit-to-stand motions (*60*–*64*). Such limitations in the force-generating capacities of the distal limb are partly attributed to optimization for swing phase costs, achieved by concentrating limb mass proximally and decreasing the moment of inertia (*65*, *66*).

The shortfall of force-generating capacities became even more pronounced in the giant alligatoroid *Deinosuchus* (3.7 tonnes). When *F*_max_ of all muscles in *Deinosuchus* was scaled isometrically (∝ mass^0.67^) from the base model, and the animal was modeled as moving in a manner dynamically similar to the adult alligator (140 kg), all major joint motions were significantly deficient in muscle forces to counteract external joint moments, with the ratio of reserve actuator moment to corresponding external joint moment exceeding 0.2 (Fig. 3A and fig. S4A). To follow the recommendation of Hicks *et al.* (*48*) and keep the ratio of reserve actuator moments to corresponding external joint moments closer to 5% for these joint motions (except ankle plantarflexion), *F*_max_ needed to be scaled with an exceptionally strong positive allometry (∝ mass^0.8^). This would result in a 2.37-fold increase in *F*_max_ and masses of hindlimb muscles, as compared to isometric scaling of *F*_max_ (∝ mass^0.67^), without altering muscle fiber lengths. We consider such allometry unlikely, as extant crocodylians exhibit near-isometric scaling of physiological cross-sectional area (proportional to *F*_max_) for most muscles (*50*). It is also worth noting that the femur length is slightly shorter than expected for the total body length in *Deinosuchus* (TMM43632-1), based on the scaling relationship observed in American alligators (fig. S8) (*67*). Thus the estimated *F*_max_ for *Deinosuchus* is probably conservative and unlikely to be overestimated.

In addition to reduced muscle capacities, increased bone stresses further constrain terrestrial locomotor capabilities of larger alligators and *Deinosuchus* (Fig. 4A). While the midshaft axial tensile stress of the femur remained well below the yield (78.6 MPa) and ultimate (108 MPa) stresses (*44*, *68*) in juveniles (0.2 to 5.6 kg), it approached the yield stress in the adult (140 kg), resulting in a safety factor for midshaft axial tension of 1.4 to 1.92 (1.8 to 2.47 when the initial CoP was shifted closer to the ankle). Calculated estimates of safety factor for the femur of *Deinosuchus* (3.7 tonnes) were even lower, ranging from 0.49 to 0.68 for our baseline analysis to 0.82 to 1.12 when the initial CoP was shifted closer to the ankle. Rather than interpreting safety factors below one as evidence of bone failure, we view such low safety factors as indicative of substantially limited terrestrial locomotor capabilities in this exceptionally large crocodylian. This safety factor estimate is minimally sensitive to changes in the *F*_max_ scaling (fig. S6B). Moreover, the “quasistatic” femoral midshaft axial tensile stress, calculated at the instant of peak bone stresses of the *Deinosuchus* simulation, was only 3% smaller than the stress originally calculated with segment acceleration, indicating that the effect of segment acceleration on bone stress is negligible. Another factor to consider is limb posture, because adopting more erect limbs reduces size-specific muscle forces and mitigates bone stresses, demonstrated by size-related posture changes in mammals and birds across species (*32*, *33*, *35*, *36*) and American alligators throughout ontogeny (*39*, *40*). However, the extent to which limb posture changes are still possible beyond the size of adult alligators remains uncertain. In mammals larger than horses (~300 kg mass), the capacity for further limb extension appears to be limited (*69*–*72*). Moreover, computational musculoskeletal simulations of the alligatorid *Caiman crocodilus* indicated that thigh muscle fiber lengths become suboptimal when extremely erect and upright limb postures are adopted (*73*), a constraint on limb posture that may have similarly affected *Deinosuchus*. Other factors, such as changes in muscle architecture, bone material properties, and gait, may also contribute to reducing bone stresses. Nonetheless, given that distal limb bones like the tibia typically exhibit even lower safety factors than the femur in alligators and iguanas (*44*), it is likely that *Deinosuchus* had minimal, if any, ability to lift its body off the ground and walk, based on the magnitudes of limb bone loads predicted by our model. The critical body size at which alligators and *Deinosuchus* become unlikely to walk would lie between the size of the adult alligator (140 kg) and that of *Deinosuchus* (3.7 tonnes). This size threshold may be even lower without the size-related posture changes observed in alligators.

It remains unclear whether the limited terrestrial locomotor capabilities of adult *Deinosuchus* are related to its extensive use of aquatic habitats. While *Deinosuchus* is known to have inhabited coastal plains to near-shore environments, as evidenced by its occurrences in Campanian deposits along the coast of the Western Interior Seaway in North America (*74*–*76*), its potential use of open-water habitats has not been confirmed (*74*). Moreover, multiple trace fossils suggest interactions with dinosaurs, either through predation or scavenging, implying a habitat near land (*74*, *77*, *78*). Conceivably, *Deinosuchus* gradually reduced its terrestrial locomotor capabilities through ontogeny, ultimately losing the ability to lift its body off the ground before reaching full size. In large *Deinosuchus*, belly-dragging may have been the primary mode of locomotion on land, with the torso and tail supporting a substantial portion of the body mass (*79*, *80*).

### Posture-related changes in hindlimb muscle activations and femoral stresses

If adopting more erect hindlimb postures helps modulate mass-specific muscle forces and maintain bone stresses in larger alligators, muscle activations and bone stresses should increase with more sprawling (abducted) hip postures among individuals of similar sizes. Our locomotor simulation of small juveniles (0.23 to 0.25 kg) partially supported this prediction, by showing increased activations of hip adductors (puboischiofemoralis externus 2 and 3) with more sprawling (abducted) hip postures (Fig. 3C, fig. S5, and table S3). However, other primary antigravity muscles did not show posture-related changes in activations, with some ankle plantarflexors even showing reduced activations with more abducted hip postures (Fig. 3C and table S3). Therefore, it remains unclear whether adopting more erect postures can reduce antigravity muscle activation in alligators.

Despite the ambiguous trends in muscle activations, bone stresses exhibited clear posture-related patterns: Both axial and torsional stresses at the femoral midshaft increased with more abducted hip postures (Fig. 5). Femoral midshaft stresses are determined by knee joint reaction forces and moments, which largely reflect the ground reaction force, along with muscle, gravitational, and inertial forces acting on the distal femoral segment (fig. S2) (*81*, *82*). Considering that the activations of muscles exerting forces on the distal femur, such as the knee extensors (e.g., iliotibialis and femorotibialis) and the gastrocnemius, were unaffected by limb posture (Fig. 3C and table S3) and that contributions from gravitational and inertial forces were small, posture-related changes in bone stresses are likely attributable to variations in the ground reaction force. Specifically, with more abducted hip postures, the ground reaction force is nearly perpendicular to the femoral long axis, thereby increasing both torsional moments (*83*, *84*) and bending moments around the femoral midshaft, leading to greater overall bone stresses.

The proposed mechanism for posture-related changes in femoral stress, driven by the relative orientation of the ground reaction force to the femur, differs from the previously suggested mechanism driven by muscle forces. Based on experimental data from alligators, it was hypothesized that adopting more erect postures causes the center of pressure to shift away from the ankle, increasing the ankle dorsiflexion moment from the external force, hence requiring greater forces from ankle plantarflexors (e.g., gastrocnemius externus) (*45*, *85*). Because the gastrocnemius externus crosses the knee joint and inserts on the ventral side of the lateral femoral epicondyle, it exerts a knee flexion moment that must be counteracted by knee extensor muscles (e.g., femorotibialis and iliotibialis). These muscles generate a moment to bend the distal femur dorsally, augmenting the bending moment from the ground reaction force and increasing stresses on the femur (*45*, *85*). The current data, lacking reliable CoP data, do not allow for direct testing of this hypothesis. Sensitivity analysis with different CoP positions revealed mixed CoP-related trends in bone stresses, with a general decrease in peak midshaft axial tensile and compressive stresses but a general increase in peak torsional stress when the initial CoP was shifted closer to the ankle (table S4). The slight reduction (10 to 12%) in peak midshaft axial tensile and compressive stresses with the ankleward (proximal) shift in CoP may align with the idea that changes in bone stresses result from a complex interplay of altered limb posture, CoP, and muscle function (*45*, *85*). However, despite the substantial displacement in the initial CoP (~^1^/_4_ of foot length) in the sensitivity analysis, its effect was less pronounced than about a 50% change in peak midshaft axial tensile stress observed across different hip adduction angles in small juveniles (Fig. 5A).

If adopting more erect limb postures reduces limb bone stresses, then it would relax biomechanical constraints, facilitating the evolution of larger body sizes. Testing this prediction using the fossil record is not straightforward, as the evolution of body size was constrained by various biological factors including musculoskeletal anatomy and physiology, as well as external factors such as resource availabilities, intra- and interspecific competition, and climate (*86*–*88*). Nevertheless, the transitions from more sprawling to more erect limb postures appear to have increased the upper size limits of tetrapods. The body size of terrestrial tetrapods with nonerect limbs is capped at around 1 tonne, with the largest taxa being sprawling pareiasaurs (e.g., *Bradysaurus* and *Scutosaurus*) and “semi-erect” dinocephalian therapsids (e.g., *Tapinocaninus* and *Jonkeria*) in the Permian (*89*–*94*). Multitonne giants (*71*) did not evolve until the emergence of the erect-limbed dicynodont *Lisowicia* and early sauropodomorph dinosaurs (e.g., *Lessemsaurus*) in the Late Triassic (*95*, *96*). Jurassic and Cretaceous sauropodomorphs eventually attained the largest size for terrestrial animals (up to ~70 tonnes) (*97*, *98*), which would not have been possible without erect, columnar limbs.

## MATERIALS AND METHODS

### Hindlimb kinematics, kinetics, and musculoskeletal model

Eight juvenile to adult American alligators were used for measuring hindlimb kinematics and ground reaction forces (table S1) (*39*, *40*). The experimental protocols were approved by the Royal Veterinary College CRERB (URN 2022 2096-3) and Clemson University IACUC (AUP 2021-070). A base musculoskeletal model was generated for the right hindlimb of a juvenile (al05: total length, 1.28 m; mass, 5.64 kg). Joint coordinate systems (JCSs) for the hip, knee, and ankle were established following the protocol outlined by Gatesy *et al.* (*99*). The hip was modeled with three rotational degrees of freedom (hip extension-flexion and abduction-adduction, and femoral long-axis rotation), while the knee and ankle were modeled with one rotational degree of freedom (extension-flexion) each. The neutral pose (reference; all angles at 0°) was set with the hindlimb splayed laterally. Thirty-six hindlimb musculotendon actuators were modeled using a Hill-type model with force-length-velocity relationships (table S2) (*100*, *101*). Muscle paths were reconstructed in OpenSim 4.4 (*102*) using via points and wrapping objects, based on magnetic resonance imaging (MRI) data. Detailed methodology for data acquisition and processing of hindlimb kinematics and kinetics, as well as musculoskeletal model generation, is provided in the Supplementary Materials.

### Musculotendon properties

Hindlimb muscle properties of the base model (al05: total length, 1.28 m; mass, 5.64 kg), including the resting fiber lengths, pennation angles, and belly masses, were adopted and scaled based on measurements from a similarly sized individual (specimen 7: total length, 1.26 m; mass, 5.1 kg) (*49*). The maximal isometric force (*F*_max_) for each muscle was estimated as

$$F_{\text{max}}=\frac{m_{\text{musc}}\cdot \sigma }{\rho \cdot l_{0}\;}$$ (1)

where *m*_musc_ is muscle belly mass, σ is maximum fiber stress (300,000 N m^−2^) (*103*–*105*), ρ is muscle density (1060 kg m^−3^) (*106*), and *l*_0_ is optimal fiber length. The calculation assumes that the resting fiber length represents optimal fiber length. Note that pennation angle was not included in the *F*_max_ calculation, as it is inherently accounted for in the geometric underpinnings of the Hill-type model in OpenSim software (*100*, *101*). Tendon slack length, another required parameter in the Hill-type model (*100*, *101*), was estimated using a modified approach from Manal and Buchanan (*107*–*109*). Details of the estimation and tuning of tendon slack length are provided in the Supplementary Materials.

### Model scaling

Given the labor-intensive nature of creating subject-specific musculoskeletal models, we scaled the body mass and segment proportions of the base model to generate individual models of different sizes in OpenSim. Each individual was assigned a body mass, and predetermined scale factors were applied to the body, thigh, shank, and foot segments on the basis of the scaling relationships of the total body, femur, tibia, and metatarsal III lengths in American alligators (*67*, *110*). Muscle-tendon unit properties in the individual models were also scaled from the base model. Maximal isometric force was scaled as mass^0.67^, given the overall isometric scaling of physiological cross-sectional area with body mass in American alligators (*49*). Muscle fiber and tendon slack lengths were scaled with the length of the primary segment (body, thigh, shank, or foot) associated with each muscle (table S2).

### Joint kinematics, dynamics, and muscle activation

Stance-phase joint angles were estimated using inverse kinematics in OpenSim, which minimizes the distance between experimentally recorded skin markers and the corresponding model markers manually placed at each joint. The body roll was locked at the ground-body joint in the pelvis, as experimental markers placed exclusively on the right side of the body may not accurately capture it. The knee and ankle joints were constrained to rotation around their extension-flexion axis. The positions of the model markers were iteratively adjusted to minimize body and segment wiggling. Stance-phase joint moments were estimated using inverse dynamics in OpenSim. Joint angles estimated through inverse kinematics and experimentally measured ground reaction forces were used to calculate joint moments. Joint moments were normalized to N·m kg^–4/3^ to enable comparisons across individuals of different body sizes. The CoP was fixed below the marker on the metatarsophalangeal joint of digit III, roughly halfway between the toe-tip and ankle, during the first half of stance. In the second half of stance, the CoP was shifted constantly toward the toe-tip, reaching it at the end of stance. To assess the sensitivity of the results to the CoP, we repeated all analyses with the initial CoP shifted closer to the ankle, positioned below the midpoint between the markers on the ankle and the metatarsophalangeal joint of digit III.

Stance-phase muscle activations and forces were estimated using static optimization in OpenSim. Given a model, joint angles filtered at 5 Hz, gravitational and inertial forces, and ground reaction forces, static optimization minimizes the sum of squared muscle activations (scaled from 0 to 1) at each time step, ignoring activation dynamics and tendon compliance (*48*). Muscle-tendon unit forces were calculated using intrinsic force-length-velocity properties (*101*). Residual force actuators were applied to the body segment, and residual torque actuators were added to pitch and yaw axes of the ground-body JCS in the pelvis (*48*). Additionally, custom reserve torque actuators were added to the hip, knee, and ankle joints to account for ignored passive structures and insufficient muscle forces (*48*). Optimal reserve actuator torques were set significantly lower than the estimated joint moments to impose high cost on their activations. These torques were scaled as *m*^4/3^·*g*/2000 for hip extension-flexion and abduction-adduction, as well as knee and ankle extension-flexion, and *m*^4/3^·*g*/4000 for the femoral axial rotation, where *m* is mass in kilograms and *g* is gravitational acceleration. Ideally, these values should remain below 5% of the corresponding external joint moments during locomotion (*48*). The default and minimum activations of each muscle were set to 0.05 and 0.01, respectively.

### Bone stress

Stance-phase bone stresses at the femoral midshaft were estimated from the net force and moment acting on and about the midshaft, along with the cross-sectional geometries of the midshaft (fig. S2) (*111*). First, a midshaft joint was added to the femur, and the midshaft JCS was established. The midshaft joint coordinate was determined by slicing the femoral shaft between the hip and knee joints, drawing a line connecting the centroid of the slices, and locating its midpoint (*112*, *113*). The midshaft *x* axis was defined as the line connecting the centroids of the midshaft slice and the adjacent distal slice. The midshaft *y* axis (mediolateral axis) was derived as the cross product of the midshaft *x* axis and knee *y* axis (knee dorsoventral axis; not shown in Fig. 1A), originating from the midshaft coordinate. The midshaft *z* axis (dorsoventral axis) was perpendicular to both the midshaft *x* and *y* axes. The net midshaft joint forces and moments were calculated using joint reaction analysis in OpenSim. Using a model, with joint angles filtered at 5 Hz, ground reaction forces, and muscle forces estimated through static optimization, joint reaction forces and moments were computed recursively from the distal to proximal segments by balancing forces acting on each segment (*81*, *82*). The femoral midshaft joint reaction forces and moments were derived from the knee joint reaction forces and moments, as well as muscle, gravitational, and inertial forces acting on the distal femoral segment. Muscle forces from those attaching to or crossing the distal femoral segment were included in the calculations.

Normal bending stress (σ_bend_) and axial stress (σ_ax_) were calculated as

$$\sigma_{\text{bend}}=\sqrt{\;{\left(\frac{M_{y}\cdot y}{I_{y}}\right)}^{2}+{\left(\frac{M_{z}\cdot z}{I_{z}}\right)}^{2}\;}$$ (2)

$$\sigma_{\text{ax}}=\frac{F_{x}}{A}$$ (3)

where *y* is the mean perpendicular distance from the dorsalmost and ventralmost cortices to the midshaft *y* axis, and *z* is the mean perpendicular distance from the medialmost and lateralmost cortices to the midshaft *z* axis. *M_y_* and *M_z_* are the moments about the midshaft *y* and *z* axes, respectively. *I_y_* and *I_z_* are the second moment of area about the midshaft *y* and *z* axes, respectively. *F_x_* is the axial force, and *A* is the section area. Positive σ_ax_ indicates axial tension, while negative σ_ax_ indicates axial compression. The slope of the neutral axis of bending (φ_na_) was then determined as

$$\varphi_{\text{na}}=\text{atan}\left(\frac{M_{z}}{M_{y}}\right)$$ (4)

A zero φ_na_ indicates that the neutral axis is aligned with the midshaft *y* axis (mediolateral axis), whereas positive φ_na_ (<π/2) indicates a counterclockwise rotation of the neutral axis in the distal view of the cross-section. Axial tensile stress (σ_tens_) and axial compressive stress (σ_comp_) were calculated as

$$\sigma_{\text{tens}}=\sigma_{\text{bend}}+\sigma_{\text{ax}}$$ (5)

$$\sigma_{\text{comp}}=-\sigma_{\text{bend}}+\sigma_{\text{ax}}$$ (6)

Torsional stress (σ_tor_) was computed as

$$\sigma_{\text{tor}}=\frac{M_{x}\cdot \left(y+z\right)}{2J}$$ (7)

where *M_x_* is the moment about the midshaft *x* axis and *J* is the polar second moment of area about the midshaft *x* axis (fig. S2). Note that the bone stress calculation above assumes a circular cross-section with bone material distributed symmetrically about the midshaft *y* and *z* axes.

Cross-sectional geometries of the femoral midshaft, required for calculating bone stress, were obtained from micro-CT (μCT) scans of four cadaveric individuals used in locomotor experiments (al05, al07, al09, and al10; table S1) and three dry femora of American alligators (UF39622, UF42474, and UF98341). Cadaveric individuals were scanned at the Godley-Snell Research Center, Clemson University (Clemson, SC, USA), using a Rigaku Quantum GX2, with a peak voltage of 90 kV, tube current of 88 mA, exposure time of 240 ms, in-plane pixel resolution of 0.09 mm, and slice thickness of 0.09 mm. Dry femora were scanned at the Nanoscale Research Facility, University of Florida (Gainesville, FL, USA), using a Phoenix V|tome|x S240, with a peak voltage of 100 to 150 kV, tube current of 200 mA, exposure time of 200 to 333 ms, in-plane pixel resolution of 0.03 to 0.09 mm, and slice thickness of 0.03 to 0.09 mm.

The section modulus about the midshaft *y* axis (*I_y_*/*y*) and *z* axis (*I_z_*/*z*), along with the polar section modulus [2 *J*/(*y* + *z*)], was measured from the midshaft cross-sections in the seven individuals. The section modulus of the adult individual, “Flounder,” who participated in the locomotor experiment but for whom scan data were lacking, was provided by a dry femur of a different American alligator (UF98341). This was possible because the femur length of Flounder, estimated at 211.6 mm from using a standardized major axis regression of total length and femur length in American alligators (*67*), closely aligned with the femur length of UF98341 (210.5 mm). Section modulus data for the other three experimental individuals (al08, al11, and al12), also lacking scan data, were estimated using regression of the section modulus and femur length in available cadaveric and dry specimens.

To visualize cross-sectional stress distribution with a neutral axis of bending, subject-specific bending stress was calculated for the base model, accounting for the asymmetric bone material distribution about the *y* and *z* axes by incorporating the product of inertia into the stress calculation (*114*). Cross-sectional geometric properties were computed from digitized midshaft sections using custom Python code.

### Modeling the giant extinct alligatoroid crocodylian

A musculoskeletal model of the extinct alligatoroid crocodylian *D. riograndensis* was created by scaling the base alligator model to match the size of the adult *D. riograndensis* (TMM43632-1). First, centrum lengths of available dorsal vertebrae in *Deinosuchus* were measured, and missing centrum lengths were estimated using the average of the preceding and subsequent centra. The total length was estimated using the allometric relationship between total length and the sum of dorsal centrum lengths in extant crocodylians (*115*). On the basis of the estimated total length of 8.68 m, body mass was determined using the allometric relationship between body mass and total length in American alligators (*38*), yielding an estimated mass of 3704.5 kg. The hindlimb segment (thigh, shank, and foot) lengths in *Deinosuchus* were determined by measuring the right femur length of TMM43632-1 and applying the hindlimb segment proportions of Flounder (total length, 3.08 m; mass, 140 kg). Although hindlimb segment proportions change allometrically in extant crocodylians (*53*, *116*, *117*), it was not possible to account for such potential changes in *Deinosuchus*. This is because the experimentally measured limb joint angles in Flounder were used as inputs for the locomotor simulation in *Deinosuchus*. For inverse dynamics, static optimization, and joint reaction analysis in *Deinosuchus*, ground reaction force was scaled proportionally to mass, and stance time was scaled as mass^1/6^ on the basis of “Flounder’s” trial, following predictions based on dynamic and geometric similarities (*35*). Muscle-tendon unit properties, along with residual/reserve actuator forces and moments required for static optimization in *Deinosuchus*, were scaled from the base model in the same manner as those of other experimental individuals. To examine the effect of different *F*_max_ scaling with body mass, *F*_max_ was also scaled as the 0.7, 0.75, and 0.8 power of body mass, in addition to the isometric scaling (∝ mass^0.67^), from the base alligator model. The femoral midshaft cross-sectional properties of *Deinosuchus*, necessary for bone stress calculation, were captured from the right femur (TMM43632-1) that is broken in half.

### Model evaluation and analysis

Simulated muscle activation and bone stress patterns were evaluated (“validated”) against prior EMG and bone strain recordings. Given the static analysis here and walking speed simulated, our comparison of muscle activations (i.e., at sarcomere level) to EMG excitation data (i.e., at neuromuscular level) is justifiable. Muscle onset and offset times within a stride were identified from the static optimization results of a representative trial (al05f90), applying a cutoff activation level of 0.05. The stance-to-stride ratio (duty factor) was adjusted to 0.7 to align with EMG data (*45*–*47*). Additionally, axial stress distributions and the neutral axis of bending were compared with those from in vivo bone strain recordings (*44*). Prior in vivo bone strain data were converted to stresses using the elastic modulus of the alligator femur (12.1 GPa) (*68*).

To examine how hindlimb posture affects limb muscle activation and bone stresses, linear regressions were performed on three small juveniles of similar sizes (al10, al11, and al12; body masses, 0.23 to 0.25 kg), thereby controlling for body size. Mean midstance (0.25 to 0.75 stance) muscle activation and bone stress characteristics, including peak midshaft axial tensile stress and peak torsional stress during stance and their ratio, were regressed on mean midstance hip adduction angles. For regressions examining the relationship between muscle activation and limb posture, muscles with mean midstance activations below 0.02 were excluded. A linear mixed-effects model incorporating intercept and/or slope of individual as random effect was not used because of a tendency for overfitting. Data manipulation, analyses, and visualization were performed using R base, tidyverse, smatr, circular, and ggplot2 packages (*118*–*122*).

## Supplementary Materials

### This PDF file includes:

Supplementary Text Figs. S1 to S8 Tables S1 to S5 References
