# Supplementary material for: Biomechanical simulations of hindlimb function in Alligator provide insights into postural shifts and body size evolution
Source: Sci Adv. 2025 Oct 22;11(43):eadx3811. doi: 10.1126/sciadv.adx3811 (PMC12542966; doi:10.1126/sciadv.adx3811)
Supplement: Supplementary file 1 — Supplementary Text Figs. S1 to S8 Tables S1 to S5 References [file sciadv.adx3811_sm.pdf]

Science Advances
AAAS

Supplementary Materials for

Biomechanical simulations of hindlimb function in *Alligator* provide insights into postural shifts and body size evolution

Masaya Iijima *et al.*

Corresponding author: Masaya Iijima, miijima8@gmail.com

Sci. Adv. **11**, eadx3811 (2025)
DOI: 10.1126/sciadv.adx3811

This PDF file includes:

Supplementary Text
Figs. S1 to S8
Tables S1 to S5
References

Supplementary Text

Kinematic and external force data collection

Stance phase kinematics and external forces were previously recorded in eight American alligators weighing between 0.2 to 140 kg (Table S1) (*39*, *40*) . Six skin markers were used to capture pelvic and hindlimb kinematics: two midline markers on the pelvis (one between two rows of pelvic osteoderms and another halfway between this point and the mid-trunk), and four markers on the hindlimb (hip, knee, ankle, and metatarsophalangeal joint of digit III). Ground reaction forces were zeroed at the baseline, filtered with a low-pass Butterworth filter at a 10 Hz cutoff frequency, and then subsampled at 20–50 Hz to match the sampling rate of kinematics for each trial (*40*). Fifty-one pairs of kinematic and ground reaction force data were available in this study, after excluding trials that resulted in unsuccessful inverse kinematics.

Musculoskeletal model generation

A base model was created using one of the individuals measured for kinematics and ground reaction forces (al05: total length 1.28 m, mass 5.64 kg). Following the *in vivo* bone strain experiment (*43*), the individual was euthanized and subsequently CT scanned. Experimental procedures and ethical approval details were provided in Iijima *et al*. (*40*). Body parts (whole body excluding the right hindlimb, right thigh, shank, and foot), cavities (trachea and lung), and pelvic and hindlimb bones were segmented using Avizo 8.1 (Thermo Fisher Scientific, Waltham, MA, USA).

Joint coordinate systems (JCSs) were established for the segmented pelvis and hindlimb bones following the protocol outlined in Gatesy *et al*. (*99*). This involves creating anatomical coordinate systems (ACSs) by selecting mesh patches for each joint, including the sacral centra, hip (acetabulum and femoral head), knee (femoral condyles and tibial and fibular heads), and ankle (astragalus and calcaneal rollers). Primitives (cylinder, sphere, or plane) were then fitted to mesh patches to define the ACS origin and axes using Geomagic Wrap 2021 (3D Systems, NC, USA). Four hindlimb segments—the pelvis, thigh (femur), shank (tibia, fibula, and astragalus), and foot (calcaneum, distal tarsals, metatarsals, and phalanges)—were articulated using paired ACSs to establish JCSs at each joint in Maya 2024 (Autodesk, San Rafael, USA). We modeled the foot as a single segment, excluding the metatarsophalangeal joint, as alligators primarily use plantigrade foot postures (*37*, *85*). For the knee and ankle, joint articulations were based on the scanned hindlimb with the knee and ankle joints flexed at approximately 90°, and the foot inverted by about 30°.

The neutral pose (reference; all angles 0°) was set with the hindlimb splayed laterally and the knee and ankle joints fully extended (Fig. 1A). For the hip JCS, z (blue), y (green), and x (red) axes correspond to hip extension-flexion and abduction-adduction, and femoral long-axis rotation, respectively, with a z-y-x rotation order. For the knee and ankle JCSs, only flexion-extension (z-axis) was considered. The rotational direction for all JCS axes was defined using the right hand rule. Recent studies on joint range of motion highlighted the importance of accounting for all six joint degrees of freedom (DOFs), including translation (*11*, *134*). However, practical limitations of using multiple skin markers on each limb segment to improve kinematic measurements, along with constraints of applying more rigorous skeletal motion analyses (e.g.,

biplanar fluoroscopy of bone markers) (*135*, *136*) to large individuals, limited the ability to consider greater numbers of DOFs at each joint.

Thirty-six hindlimb musculotendon actuators were modeled using a Hill-type model with force-length-velocity relationships (*100*, *101*), excluding digital muscles that do not cross the ankle (Fig. 1B; Table S2). Hindlimb muscle homologies follow Suzuki *et al*. (*56*) and Hattori and Tsuihiji (*137*). To capture muscle paths, the hindlimb of the individual used to create the base model was MRI scanned at the Veterinary Teaching Hospital of the University of Georgia (Athens, GA, USA). The scan was performed using a Siemens Magnetom Skyra 3T scanner, with PD/T2 weighted sequences, echo time of 33 ms, repetition time of 3,400 ms, in-plane pixel resolution of 0.417 mm, and slice thickness of 3.0 mm. Individual muscles were digitally dissected in Avizo 8.1 and exported as 3D objects. After manually dissecting muscles and recording their attachment sites with reference to the literature (*56, 137–139*), muscle lines of action were estimated. This was done by slicing the muscle belly between the origin and insertion points and connecting the centroid of each slice, using the 'muscle line of action estimation' MEL script in Maya 2024 (*112*, *113*). The resultant muscle path objects, along with bones, were rotoscoped to align with the positions and orientations of the corresponding hindlimb segments in the neutral pose.

The pelvic and hindlimb bones, JCSs, body segment and cavity volumes, and muscle path objects were exported from Maya to OpenSim 4.4 (*102*), using a custom MATLAB code (*140*). A uniform fresh density of 1,060 kg m^{-3} was given to the body segments excluding cavities, based on the skeletal muscle densities in mammals (*106*). Muscle origins and insertions were adjusted, and muscle paths were constrained by using via points and wrapping objects (ellipsoid, cylinder, and torus), with reference to the imported muscle path objects in OpenSim. Attention was given to adding at least one via point in the intermediate limb segment for biarticular muscles to ensure accurate computation of joint reaction forces and moments in OpenSim.

<u>Tendon slack length estimation</u>

Tendon slack length was estimated using the modified approach based on Manal and Buchanan (*107*), considering a larger range of normalized fiber lengths ($0.5 \leq \tilde{l}_0 \leq 1.5$) and ignoring tendon compliance. This involved randomly sampling a set of three musculotendon lengths across the recorded joint range of motion for all experimental individuals, along with a corresponding set of three normalized fiber lengths. A steepest descent optimization was then applied to find a set of normalized fiber lengths associated with a converged tendon slack length (*107*). This procedure was repeated 1,000 times and the mean tendon slack length for each muscle was obtained as the initial estimate. The initial estimates for tendon slack length were incrementally adjusted by 5% until the range of normalized fiber lengths fell between 0.5 and 1.5. If adjusting tendon slack length alone did not optimize the range of normalized fiber length, fiber length was also incrementally adjusted by 5% to ensure that normalized fiber length remained between 0.5 and 1.5 throughout the recorded joint range of motion. Despite the best efforts, certain ankle plantarflexors (e.g., gastrocnemius externus, fibulocalcaneus, flexor digitorum longus, and flexor hallucis longus) experienced normalized fiber lengths below 0.5, when the ankle was nearly fully plantarflexed just after touchdown or before takeoff in some trials. However, these suboptimal normalized fiber lengths should have minimal impact on our analyses of muscle activations and bone stresses, as our comparisons primarily focused on mid-stance mean or whole-stance peak

values, which are typically recorded during mid-stance. Any negative tendon slack length estimates were replaced with 5% of the fiber length. For m. ambiens 2, which originates on the proximomedial surface of the pubis and inserts on the belly of m. femorotibialis internus (*56*, *139*), the fiber length measurement was likely underestimated, resulting in an overly long tendon slack length. Consequently, its fiber length was first increased, and tendon slack length was then set to 5% of the fiber length. The mean adjustment percentages across all muscles were 20% for fiber length and 5.6% for tendon slack length, excluding cases where tendon slack length was set to 5% of the fiber length.

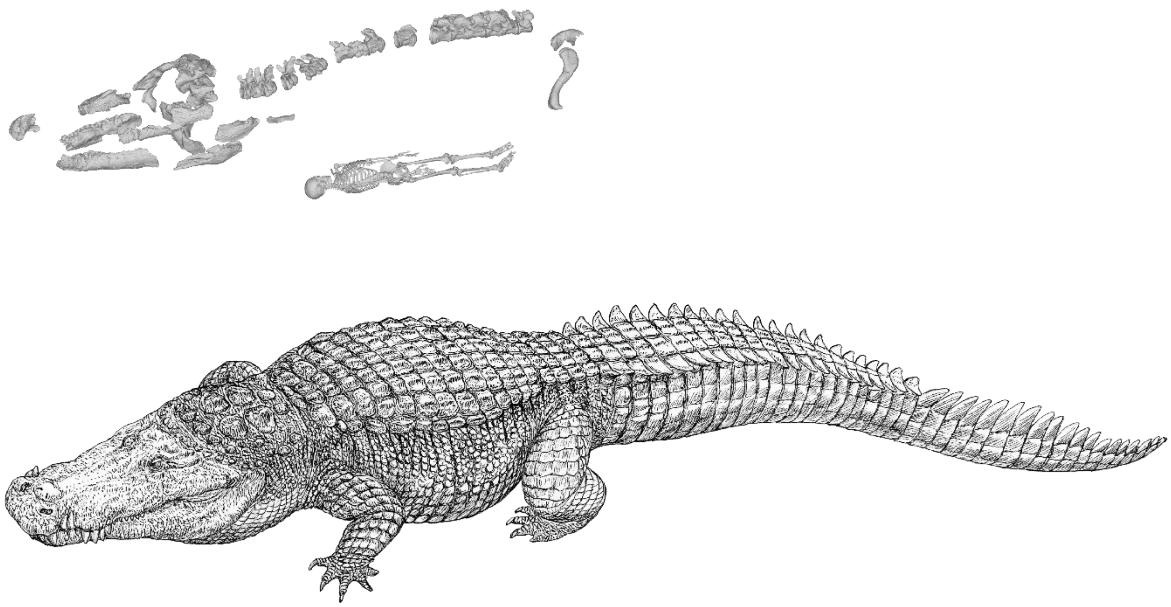

Fig. S1. Skeletal reconstruction (top) and life restoration (bottom) of *Deinosuchus riograndensis* (TMM43632-1). A 1.6 m tall human skeleton (Artec 3D: https://www.artec3d.com/3d-models/human-skeleton-hd) is shown for scale. The life restoration was drawn by Takashi Oda.

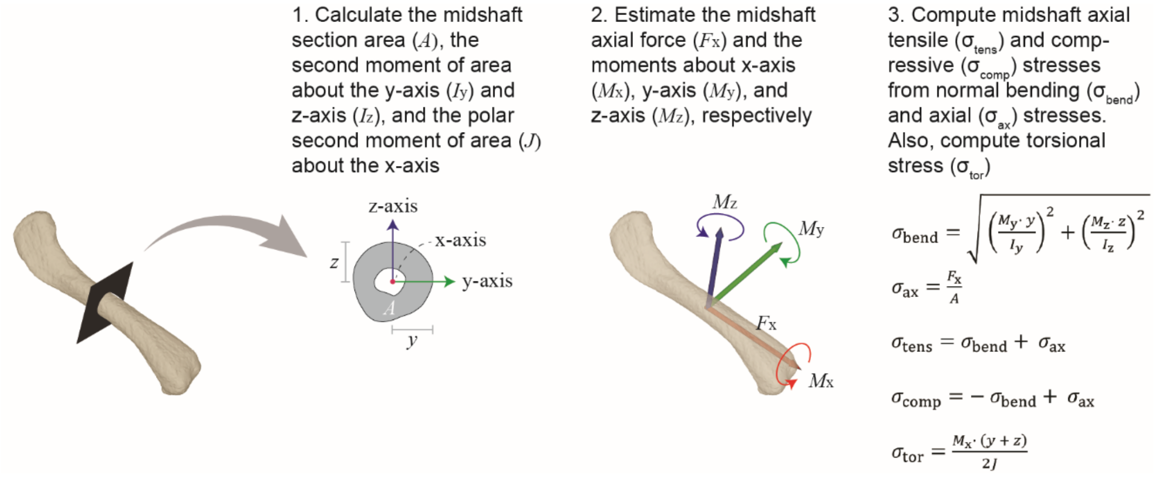

Fig. S2. Calculation of axial tensile and compressive stresses and torsional stress at the femoral midshaft.

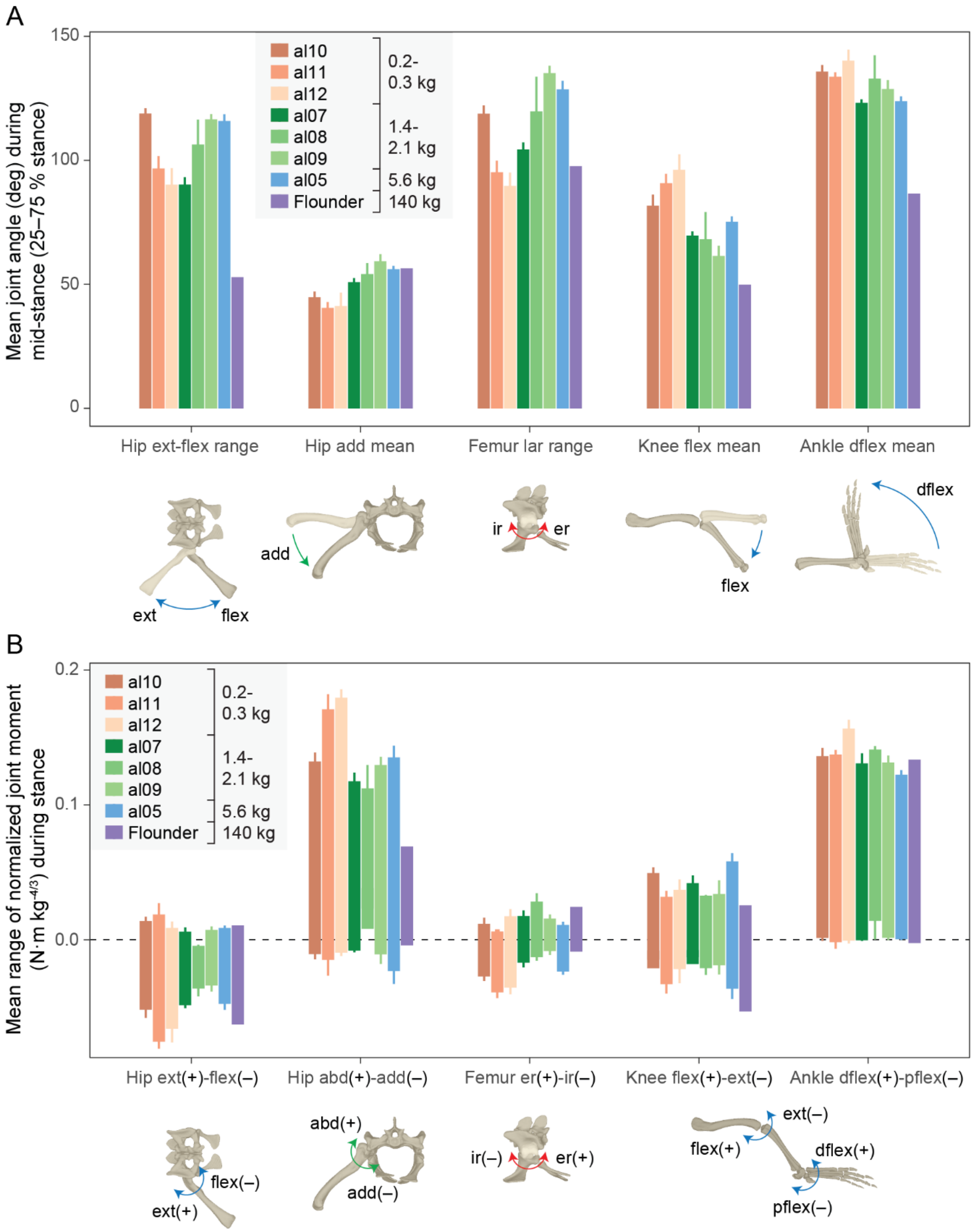

Fig. S3. Hindlimb joint angles and moments in American alligators across body sizes. (**A**) Mean joint angle (deg) during mid-stance (25–75% stance) for 8 juvenile to adult American alligators. (**B**) Mean range of normalized joint moment (N·m kg$^{-4/3}$) during stance for 8 juvenile to adult American alligators. The bar plots and error bars represent the mean ± s.e.m. Joint

motion abbreviations: add, adduction; abd, abduction; dflex, dorsiflexion; er, external rotation; ext, extension; flex, flexion; ir, internal rotation; lar, long-axis rotation; pflex, plantarflexion.

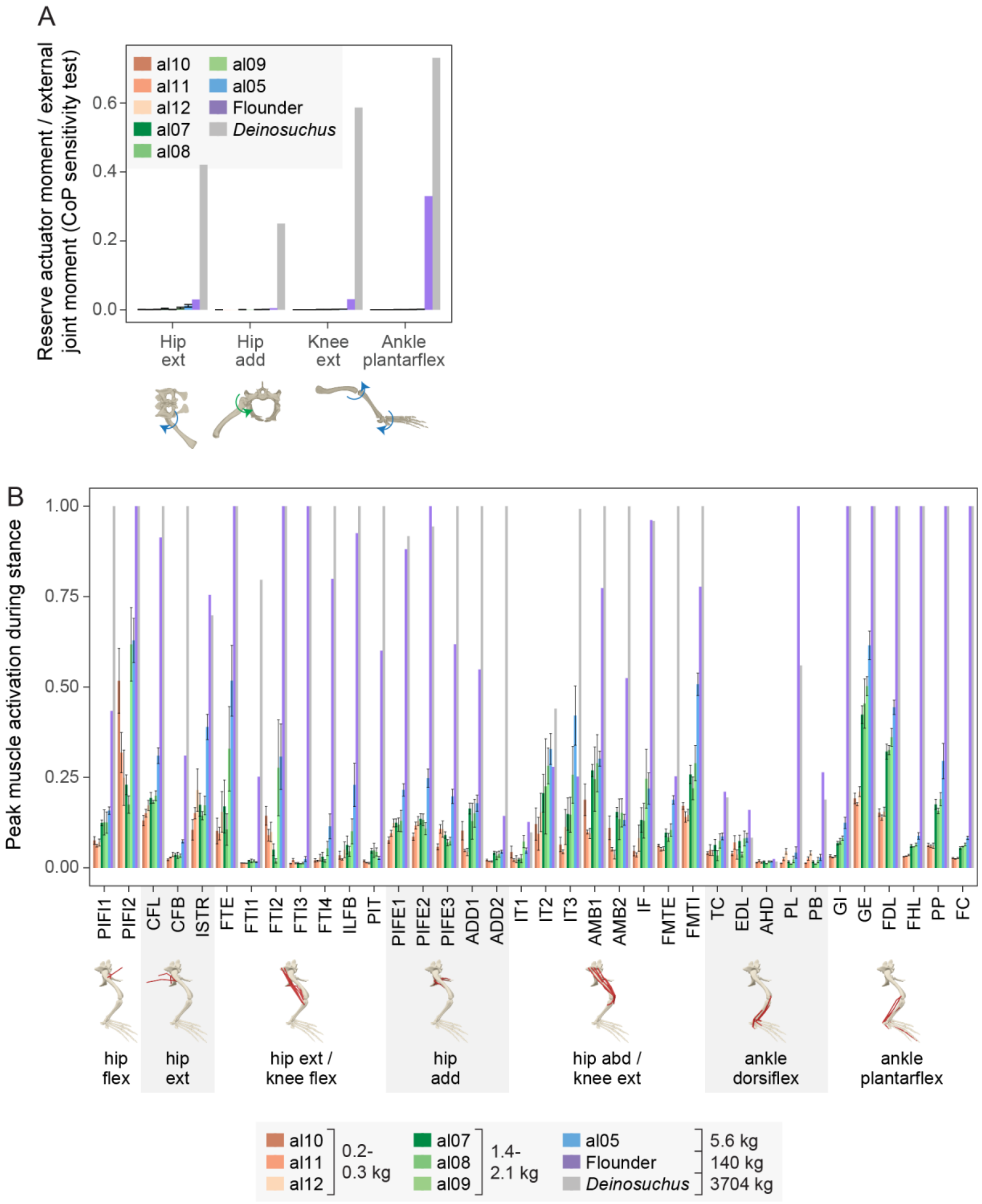

Fig. S4. Additional results on hindlimb muscle force-generating capacities in American alligators and *Deinosuchus riograndensis* across body sizes. (**A**) Ratios of reserve actuator moment to corresponding external joint moment at the instant of peak joint moment for major joint motions (hip extension and adduction, knee extension, and ankle plantarflexion) in 8 juvenile to adult American alligators and *Deinosuchus riograndensis* (TMM43632-1), computed with the initial center of pressure (CoP) shifted closer to the ankle. (**B**) Peak activations of 36

hindlimb muscles during stance in 8 juvenile to adult American alligators and *Deinosuchus riograndensis* (TMM43632-1). The bar plots and error bars represent the mean ± s.e.m. Muscle activations are scaled from 0 to 1. Full muscle names are provided in Table S2. Joint motion abbreviations: add, adduction; abd, abduction; dorsiflex, dorsiflexion; ext, extension; flex, flexion; plantarflex, plantarflexion.

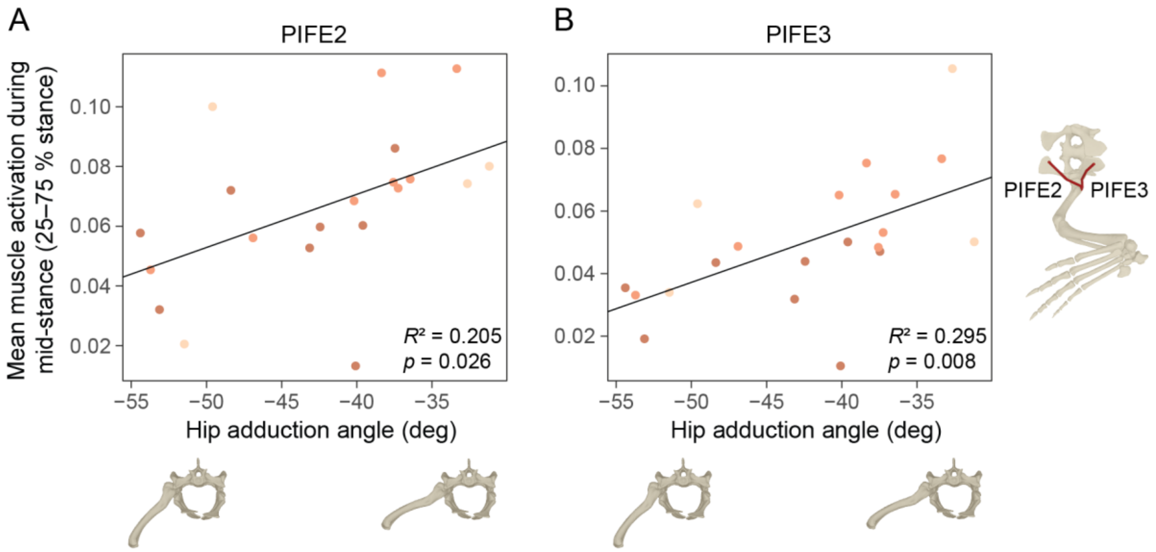

Fig. S5. Biplot of mean muscle activation during mid-stance (25–75% stance) and hip adduction angle (deg) in small juvenile alligators (al10, al11, and al12: masses 0.23−0.25 kg), with linear regression lines. (**A**) M. puboischiofemoralis externus 2. (**B**) M. puboischiofemoralis externus 3.

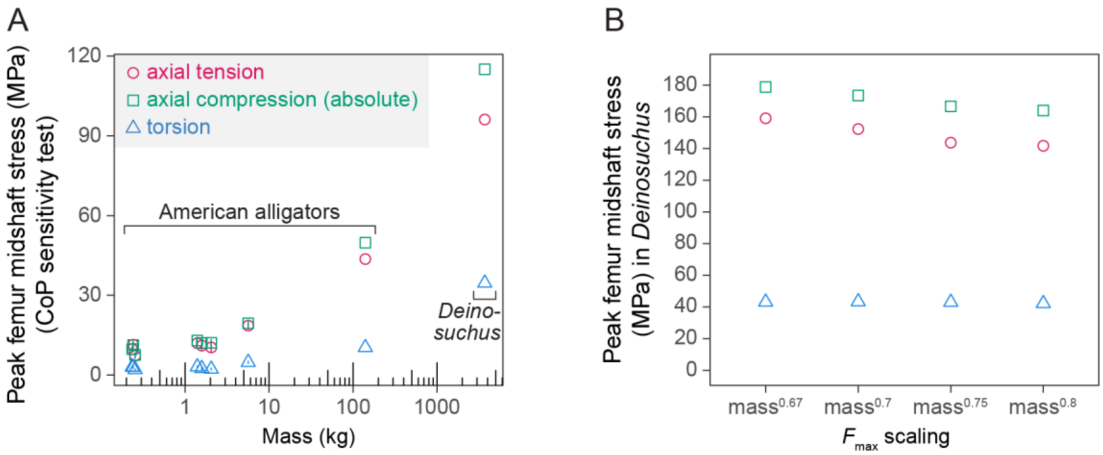

Fig. S6. Sensitivity analysis of femoral stress in American alligators and *Deinosuchus riograndensis*. (**A**) Peak femur midshaft stresses (MPa), including axial tension, axial compression (absolute values), and torsion in 8 juvenile to adult American alligators and *Deinosuchus riograndensis* (TMM43632-1), computed with the initial center of pressure (CoP) shifted closer to the ankle. Error bars represent the s.e.m. (**B**) Changes in peak femur midshaft stresses with four different F_{max} scaling factors (F_{max} scaled as the 0.67, 0.7, 0.75, and 0.8 power of body mass from the base model) for *Deinosuchus riograndensis* (TMM43632-1), computed with the default initial CoP.

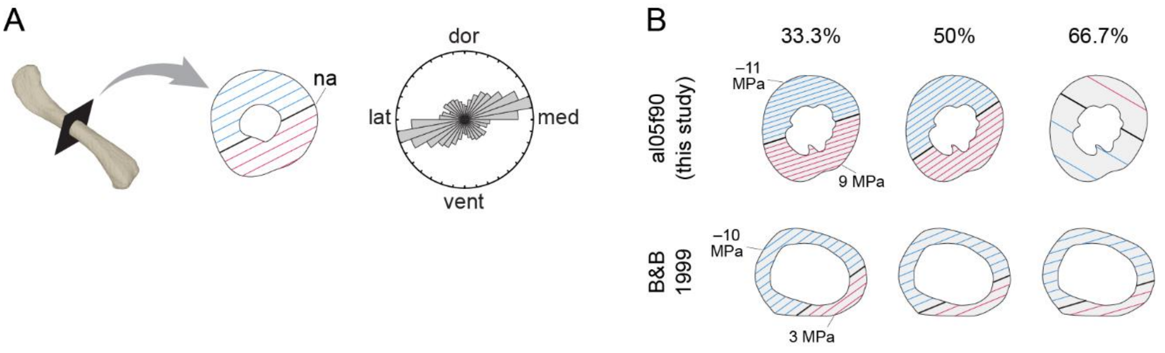

Fig. S7. Femoral midshaft axial stresses in American alligators. (**A**) Distribution of neutral axis orientations during mid-stance (25–75% stance) across all trials from 8 juvenile to adult American alligators. Neutral axis orientations were sampled at 5% intervals within 25–75% stance for each trial and plotted on the femoral midshaft cross-section from a distal perspective. (**B**) Comparisons of axial stress distribution at 33.3%, 50%, and 66.7% stance in a representative trial from a juvenile alligator (al05f90: top row) and stresses derived from *in vivo* femoral strains and the elastic modulus of the femur in a juvenile alligator (bottom row) (*44*, *68*). Stress isoclines were drawn at 1 MPa intervals, with red lines representing axial tension and blue lines representing axial compression. "B&B 1999" denotes Blob and Biewener (*44*).

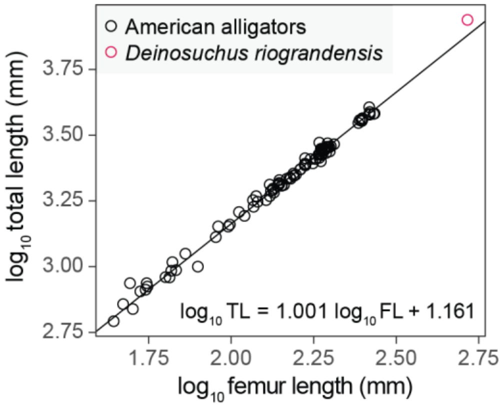

Fig. S8. Biplot of $\log_{10}$-transformed total and femur lengths (mm) in American alligators and *Deinosuchus riograndensis*, with the standard major axis regression line for American alligators. Length measurements for American alligators were adopted from Farlow *et al*. (*67*). The total length of *D. riograndensis* (TMM43632-1) was estimated based on centrum lengths of dorsal vertebrae (*115*). FL, femur length; TL, total length.

Table S1. Body lengths and masses of eight American alligators used in this study.

Individual ID	Number of trials	Total length (m)	Mass (kg)
al10	8	0.48	0.25
al11	8	0.47	0.23
al12	4	0.48	0.24
al07	8	0.81	1.40
al08	2	0.86	1.58
al09	7	0.90	2.04
al05	13	1.26	5.64
Flounder	1	3.08	140

Table S2. Muscle-tendon unit properties of the base model (al05: total length 1.26 m, mass 5.64 kg). Belly masses, pennation angles, and fiber lengths were adopted and scaled based on measurements from a similarly sized individual (specimen 7: total length 1.26 m, mass 5.1 kg) (*49*). Asterisk for tuned tendon slack length indicates adjustment by $l_0 \cdot 0.05$.

Muscle	Muscle abbreviation	Primary segment	Belly mass, m_{musc} (kg)	Pennation angle, α_0 (deg)	Measured fiber length, l_0 (m)
Puboischiofemoralis internus 1	PIFI1	trunk	0.0073	0	0.0504
Puboischiofemoralis internus 2	PIFI2	trunk	0.0305	0	0.0704
Caudofemoralis longus	CFL	trunk	0.1011	18	0.1004
Caudofemoralis brevis	CFB	trunk	0.0062	0	0.0420
Puboischiofemoralis externus 1	PIFE1	trunk	0.0076	0	0.0363
Puboischiofemoralis externus 2	PIFE2	trunk	0.0059	25	0.0252
Puboischiofemoralis externus 3	PIFE3	trunk	0.0035	22	0.0194
Ischiotrochantericus	ISTR	trunk	0.0018	39	0.0102
Adductor femoris 1	ADD1	thigh	0.0055	0	0.0725
Adductor femoris 2	ADD2	thigh	0.0019	0	0.0675
Flexor tibialis externus	FTE	thigh	0.0121	0	0.0670
Flexor tibialis internus 1	FTI1	thigh	0.0010	0	0.0674
Flexor tibialis internus 2	FTI2	thigh	0.0065	0	0.0769
Flexor tibialis internus 3	FTI3	thigh	0.0036	0	0.0502
Flexor tibialis internus 4	FTI4	thigh	0.0022	0	0.0571
Iliofibularis	ILFB	thigh	0.0031	0	0.0609
Puboischiotibialis	PIT	thigh	0.0028	0	0.0643
Iliotibialis 1	IT1	thigh	0.0016	0	0.0478
Iliotibialis 2	IT2	thigh	0.0111	30	0.0260
Iliotibialis 3	IT3	thigh	0.0026	22	0.0232
Ambiens 1	AMB1	thigh	0.0048	0	0.0370
Ambiens 2	AMB2	thigh	0.0011	0	0.0178
Iliofemoralis	IF	thigh	0.0056	21	0.0271
Femorotibialis externus	FMTE	thigh	0.0031	25	0.0156
Femorotibialis internus	FMTI	thigh	0.0087	40	0.0189
Tibialis cranialis	TC	shank	0.0045	9	0.0414
Extensor digitorum longus	EDL	shank	0.0035	0	0.0522
Adductor hallucis dorsalis	AHD	foot	0.0004	0	0.0198
Peroneus longus	PL	shank	0.0014	30	0.0107
Peroneus brevis	PB	shank	0.0008	21	0.0071
Gastrocnemius internus	GI	shank	0.0033	13	0.0456
Gastrocnemius externus	GE	shank	0.0084	29	0.0157
Flexor digitorum longus	FDL	foot	0.0033	23	0.0109
Flexor hallucis longus	FHL	foot	0.0013	26	0.0145
Pronator profundus	PP	shank	0.0031	28	0.0091
Fibulocalcaneus	FC	shank	0.0005	0	0.0073

Muscle	Tuned fiber length, l_0 (m)	Estimated tendon slack length, L_s (m)	Tuned tendon slack length, L_s (m)	Max isometric force, F_{max} (N)	Remarks
Puboischiofemoralis internus 1	0.0403	−0.0040	0.0020*	40.8180	
Puboischiofemoralis internus 2	0.0422	−0.0169	0.0021*	122.5366	
Caudofemoralis longus	0.1004	0.0507	0.0507	284.9306	
Caudofemoralis brevis	0.0420	0.0225	0.0248	41.5262	
Puboischiofemoralis externus 1	0.0363	0.0269	0.0242	59.4332	
Puboischiofemoralis externus 2	0.0252	0.0321	0.0369	66.4768	
Puboischiofemoralis externus 3	0.0194	0.0323	0.0323	50.6809	
Ischiotrochantericus	0.0102	0.0366	0.0366	50.9594	
Adductor femoris 1	0.0471	−0.0314	0.0024*	21.5516	
Adductor femoris 2	0.0506	−0.0280	0.0025*	8.1180	
Flexor tibialis externus	0.0670	0.0286	0.0286	51.2215	
Flexor tibialis internus 1	0.0674	0.0246	0.0246	4.1812	
Flexor tibialis internus 2	0.0769	0.0212	0.0212	24.0646	
Flexor tibialis internus 3	0.0502	0.0393	0.0354	20.4591	
Flexor tibialis internus 4	0.0571	0.0350	0.0385	10.7482	
Iliofibularis	0.0609	0.0403	0.0444	14.6022	
Puboischiotibialis	0.0643	0.0251	0.0201	12.2230	
Iliotibialis 1	0.0478	0.0183	0.0201	9.7606	
Iliotibialis 2	0.0260	0.0872	0.0872	120.9142	
Iliotibialis 3	0.0371	0.0926	0.0833	32.1222	
Ambiens 1	0.0370	0.0667	0.0667	36.6440	
Ambiens 2	0.0463	0.0318	0.0023*	17.2394	
Iliofemoralis	0.0271	0.0164	0.0164	58.5808	
Femorotibialis externus	0.0156	0.0510	0.0510	56.2018	
Femorotibialis internus	0.0189	0.0423	0.0423	130.0543	
Tibialis cranialis	0.0414	0.0685	0.0685	30.7074	Extensor digitorum longus: Allen *et al*. (*49*)
Extensor digitorum longus	0.0522	0.0180	0.0126	18.7155	Tibialis anterior: Allen *et al*. (*49*)
Adductor hallucis dorsalis	0.0188	0.0028	0.0028	6.1676	Extensor hallucis longus: Allen *et al*. (*49*)
Peroneus longus	0.0107	0.0520	0.0520	38.0431	Fibularis longus: Allen *et al*. (*49*)
Peroneus brevis	0.0085	0.0189	0.0179	29.9894	Fibularis brevis: Allen *et al*. (*49*)
Gastrocnemius internus	0.0456	0.0525	0.0525	20.5316	
Gastrocnemius externus	0.0220	0.0978	0.0978	150.9778	
Flexor digitorum longus	0.0180	0.1535	0.1535	86.7555	
Flexor hallucis longus	0.0189	0.1701	0.1701	25.2659	
Pronator profundus	0.0155	0.0756	0.0718	96.0018	Interosseus cruris: Allen *et al*. (*49*)
Fibulocalcaneus	0.0183	0.0092	0.0064	18.4443	Pronator profundus: Allen *et al*. (*49*)

Table S3. Linear regressions of mean hindlimb muscle activation on mean hip adduction angles during mid-stance in small juvenile alligators (0.23−0.25 kg). Hindlimb muscles with a mean activation greater than 0.02 during mid-stance were included in the analysis. Asterisks indicate significant results ($p < 0.05$).

Dependent variable (muscle activation)	Observations	Intercept	Slope	*p*-value	Adjusted R^2
PIFI1	20	0.02554	−0.00025	0.608	−0.040
PIFI2	20	−0.01461	−0.00156	0.098	0.097
CFL	20	0.12581	0.00127	0.082	0.112
ISTR	20	0.09459	0.00137	0.056	0.143
FTE	20	0.01780	−0.00012	0.883	−0.054
PIFE1	20	0.09283	0.00092	0.159	0.057
PIFE2	20	0.14177	0.00178	0.026*	0.205
PIFE3	20	0.12127	0.00168	0.008*	0.295
ADD1	20	0.01263	−0.00050	0.367	−0.008
IT2	20	0.06496	0.00032	0.867	−0.054
IT3	20	0.02760	0.00019	0.615	−0.040
AMB1	20	0.00778	−0.00122	0.216	0.033
AMB2	20	0.00925	−0.00048	0.354	−0.005
FMTE	20	0.04219	0.00008	0.796	−0.052
FMTI	20	0.12068	0.00043	0.646	−0.043
TC	20	0.04983	0.00063	0.014*	0.252
GI	20	0.02165	−0.00010	0.471	−0.025
GE	20	0.12417	−0.00069	0.521	−0.031
FDL	20	0.06994	−0.00131	0.087	0.107
FHL	20	0.02361	−0.00006	0.618	−0.041
PP	20	0.01562	−0.00086	0.012*	0.263
FC	20	0.01644	−0.00015	0.121	0.080

Table S4. Peak axial tensile, compressive, and torsional stresses (MPa) and the ratio of peak axial tensile to torsional stress in eight American alligators and *Deinosuchus riograndensis*. Results are presented for both the analysis with default initial center of pressure (CoP) and sensitivity analysis with the initial CoP shifted closer to the ankle. Data are means±s.e.m.

Species	Individual ID	Number of trials	Mass (kg)	Peak axial tensile stress (MPa)	Peak axial compres-sive stress (MPa)	Peak torsional stress (MPa)	Ratio of peak axial tensile to torsional stress
**Default initial CoP**							
Alligator mississippiensis	al10	8	0.25	7.7±1.2	−8.3±1.3	1.4±0.1	5.9±1.5
Alligator mississippiensis	al11	8	0.23	8.9±0.6	−9.1±0.5	2.1±0.2	4.4±0.3
Alligator mississippiensis	al12	4	0.24	10.0±0.8	−10.3±0.8	1.8±0.3	5.9±1.1
Alligator mississippiensis	al07	8	1.40	12.9±1.3	−14.2±1.5	2.3±0.3	6.2±0.8
Alligator mississippiensis	al08	2	1.58	12.4±2.5	−13.9±2.7	2.3±0.0	5.3±1.0
Alligator mississippiensis	al09	7	2.05	10.7±1.3	−12.8±1.1	2.0±0.2	5.8±1.2
Alligator mississippiensis	al05	13	5.64	18.7±1.2	−20.3±1.1	3.3±0.3	6.3±0.7
Alligator mississippiensis	Flounder	1	140	56.2	−63.5	13.7	4.1
Deinosuchus riograndensis	TMM43632-1	1	3705	159.0	−179.0	43.1	3.7
**Initial CoP shifted**							
Alligator mississippiensis	al10	8	0.25	7.3±0.9	−7.5±0.8	2.1±0.2	4.2±1.3
Alligator mississippiensis	al11	8	0.23	7.5±1.2	−7.6±1.1	2.9±0.2	2.6±0.4
Alligator mississippiensis	al12	4	0.24	11.4±1.4	−11.3±1.3	2.9±0.4	4.3±0.9
Alligator mississippiensis	al07	8	1.40	12.0±1.2	−12.9±1.3	3.1±0.3	4.0±0.4
Alligator mississippiensis	al08	2	1.58	11.1±1.4	−12.1±0.9	2.5±0.7	5.1±2.1
Alligator mississippiensis	al09	7	2.05	10.4±1.5	−12.1±1.3	2.2±0.3	5.1±0.9
Alligator mississippiensis	al05	13	5.64	18.6±1.4	−19.5±1.2	4.8±0.4	4.1±0.3
Alligator mississippiensis	Flounder	1	140	43.7	−49.8	10.3	4.2
Deinosuchus riograndensis	TMM43632-1	1	3705	96.1	−115.0	34.6	2.8

Table S5. Linear regressions of bone stress characteristics on mean hip adduction angles during mid-stance in small juvenile alligators (0.23−0.25 kg).

Dependent variable (stress characteristic)	Observations	Intercept	Slope	*p*-value	Adjusted R^2
Peak axial tensile stress (MPa)	20	18.730	0.239	<0.001	0.440
Peak axial compressive stress (MPa)	20	−18.021	-0.212	0.006	0.315
Max absolute torsional stress (Mpa)	20	3.761	0.047	0.001	0.439
Ratio of peak axial tensile to torsional stress	20	6.042	0.017	0.849	−0.053

REFERENCES AND NOTES

1. P. J. Bishop, S. E. Pierce, The fossil record of appendicular muscle evolution in Synapsida on the line to mammals: Part I—Forelimb. *Anat. Rec.* **307**, 1764–1825 (2024).

2. P. J. Bishop, S. E. Pierce, The fossil record of appendicular muscle evolution in Synapsida on the line to mammals: Part II—Hindlimb. *Anat. Rec.* **307**, 1826–1896 (2024).

3. J. F. Bonaparte, Locomotion in rauisuchid thecodonts. *J. Vertebr. Paleontol.* **3**, 210–218 (1984).

4. A. J. Charig, "The evolution of the archosaur pelvis and hindlimb: An explanation in functional terms," in *Studies in Vertebrate Evolution*, K. A. Joysey, T. S. Kemp, Eds. (Oliver & Boyd, 1972), pp. 121–155.

5. J. R. Hutchinson, S. M. Gatesy, Adductors, abductors, and the evolution of archosaur locomotion. *Paleobiology* **26**, 734–751 (2000).

6. F. A. Jenkins Jr., The postcranial skeleton of African cynodonts: Problems in the early evolution of the mammalian postcranial skeleton. *Bull. Peabody Mus. Nat. Hist.* **36**, 1–216 (1971).

7. F. A. Jenkins Jr., The functional anatomy and evolution of the mammalian humero-ulnar articulation. *Am. J. Anat.* **137**, 281–297 (1973).

8. T. S. Kemp, Stance and gait in the hindlimb of a therocephalian mammal-like reptile. *J. Zool.* **186**, 143–161 (1978).

9. T. S. Kemp, *Mammal-Like Reptiles and the Origin of Mammals* (Academic Press, 1982).

10. J. M. Parrish, Locomotor adaptations in the hindlimb and pelvis of the Thecodontia. *Hunteria* **1**, 2–35 (1986).

11. P. J. Bishop, R. J. Brocklehurst, S. E. Pierce, Intelligent sampling of high-dimensional joint mobility space for analysis of articular function. *Methods Ecol. Evol.* **14**, 569–582 (2023).

12. O. E. Demuth, E. J. Rayfield, J. R. Hutchinson, 3D hindlimb joint mobility of the stem-archosaur *Euparkeria capensis* with implications for postural evolution within Archosauria. *Sci. Rep.* **10**, 15357 (2020).

13. P. Fahn-Lai, A. A. Biewener, S. E. Pierce, Three-dimensional mobility and muscle attachments in the pectoral limb of the Triassic cynodont *Massetognathus pascuali* (Romer, 1967). *J. Anat.* **232**, 383–406 (2018).

14. J. Fröbisch, Locomotion in derived dicynodonts (Synapsida, Anomodontia): A functional analysis of the pelvic girdle and hind limb of *Tetragonias njalilus*. *Can. J. Earth Sci.* **43**, 1297–1308 (2006).

15. P. J. Bishop, S. E. Pierce, Late acquisition of erect hindlimb posture and function in the forerunners of therian mammals. *Sci. Adv.* **10**, eadr2722 (2024).

16. R. J. Brocklehurst, P. Fahn-Lai, S. Regnault, S. E. Pierce, Musculoskeletal modeling of sprawling and parasagittal forelimbs provides insight into synapsid postural transition. *iScience* **25**, 103578 (2022).

17. R. W. Blob, Evolution of hindlimb posture in nonmammalian therapsids: Biomechanical tests of paleontological hypotheses. *Paleobiology* **27**, 14–38 (2001).

18. T. Kubo, M. J. Benton, Evolution of hindlimb posture in archosaurs: Limb stresses in extinct vertebrates. *Palaeontology* **50**, 1519–1529 (2007).

19. M. Bernardi, H. Klein, F. M. Petti, M. D. Ezcurra, The origin and early radiation of archosauriforms: Integrating the skeletal and footprint record. *PLOS ONE* **10**, e0128449 (2015).

20. T. Kubo, M. J. Benton, Tetrapod postural shift estimated from Permian and Triassic trackways. *Palaeontology* **52**, 1029–1037 (2009).

21. H. Klein, S. G. Lucas, *The Triassic Tetrapod Footprint Record*, vol. 83 of *New Mexico Museum of Natural History and Science Bulletins* (New Mexico Museum of Natural History and Science, 2021).

22. D. R. Carrier, The evolution of locomotor stamina in tetrapods: Circumventing a mechanical constraint. *Paleobiology* **13**, 326–341 (1987).

23. A. F. Bennett, J. A. Ruben, Endothermy and activity in vertebrates. *Science* **206**, 649–654 (1979).

24. M. J. Benton, The origin of endothermy in synapsids and archosaurs and arms races in the Triassic. *Gondw. Res.* **100**, 261–289 (2021).

25. M. J. Benton, F. Wu, Triassic revolution. *Front. Earth Sci.* **10**, 899541 (2022).

26. J. A. Gauthier, S. J. Nesbitt, E. R. Schachner, G. S. Bever, W. G. Joyce, The bipedal stem crocodilian *Poposaurus gracilis*: Inferring function in fossils and innovation in archosaur locomotion. *Bull. Peabody Mus. Nat. Hist.* **52**, 107–126 (2011).

27. P. C. Sereno, A. B. Arcucci, Dinosaurian precursors from the Middle Triassic of Argentina: *Marasuchus lilloensis*, gen. nov. *J. Vertebr. Paleontol.* **14**, 53–73 (1994).

28. J. C. Weinbaum, Postcranial skeleton of *Postosuchus kirkpatricki* (Archosauria: Paracrocodylomorpha), from the Upper Triassic of the United States. *Geol. Soc. Lond. Spec. Publ.* **379**, 525–553 (2013).

29. S. M. Gatesy, K. P. Dial, Locomotor modules and the evolution of avian flight. *Evolution* **50**, 331–340 (1996).

30. K. Padian, L. M. Chiappe, The origin and early evolution of birds. *Biol. Rev.* **73**, 1–42 (1998).

31. C. Sullivan, "Evolution of hind limb posture in Triassic archosauriforms," in *Great Transformations in Vertebrate Evolution*, K. P. Dial, N. Shubin, E. L. Brainerd, Eds. (University of Chicago Press, 2015), pp. 107–124.

32. A. A. Biewener, Scaling body support in mammals: Limb posture and muscle mechanics. *Science* **245**, 45–48 (1989).

33. A. A. Biewener, Biomechanics of mammalian terrestrial locomotion. *Science* **250**, 1097–1103 (1990).

34. A. A. Biewener, Biomechanical consequences of scaling. *J. Exp. Biol.* **208**, 1665–1676 (2005).

35. M. A. Daley, A. Birn-Jeffery, Scaling of avian bipedal locomotion reveals independent effects of body mass and leg posture on gait. *J. Exp. Biol.* **221**, jeb152538 (2018).

36. S. M. Gatesy, A. A. Biewener, Bipedal locomotion: Effects of speed, size and limb posture in birds and humans. *J. Zool.* **224**, 127–147 (1991).

37. S. M. Reilly, J. A. Elias, Locomotion in *Alligator mississippiensis*: Kinematic effects of speed and posture and their relevance to the sprawling-to-erect paradigm. *J. Exp. Biol.* **201**, 2559–2574 (1998).

38. G. Grigg, D. Kirshner, *Biology and Evolution of Crocodylians* (CSIRO Publishing, 2015).

39. M. Iijima, V. D. Munteanu, R. M. Elsey, R. W. Blob, Ontogenetic changes in limb posture, kinematics, forces and joint moments in American alligators (*Alligator mississippiensis*). *J. Exp. Biol.* **224**, jeb242990 (2021).

40. M. Iijima, J. Darlington, V. D. Munteanu, K. A. Vliet, R. W. Blob, Scaling of gait, limb posture, forces, and inertial properties in terrestrial locomotion of American alligators across a thousand-fold increase in body mass. *Integr. Comp. Biol.* **63**, 664–680 (2023).

41. C. J. Clemente, P. C. Withers, G. Thompson, D. Lloyd, Evolution of limb bone loading and body size in varanid lizards. *J. Exp. Biol.* **214**, 3013–3020 (2011).

42. M. Iijima, C. J. Mayerl, V. D. Munteanu, R. W. Blob, Forelimb muscle activation patterns in American alligators: Insights into the evolution of limb posture and powered flight in archosaurs. *J. Anat.* **244**, 943–958 (2024).

43. M. Iijima, V. D. Munteanu, R. W. Blob, Variations in humeral and femoral strains across body sizes and limb posture in American alligators. *J. Exp. Biol.* **227**, jeb249211 (2024).

44. R. W. Blob, A. A. Biewener, *In vivo* locomotor strain in the hindlimb bones of *Alligator mississippiensis* and *Iguana iguana*: Implications for the evolution of limb bone safety factor and non-sprawling limb posture. *J. Exp. Biol.* **202**, 1023–1046 (1999).

45. S. M. Reilly, R. W. Blob, Motor control of locomotor hindlimb posture in the American alligator (*Alligator mississippiensis*). *J. Exp. Biol.* **206**, 4327–4340 (2003).

46. S. M. Gatesy, An electromyographic analysis of hindlimb function in *Alligator* during terrestrial locomotion. *J. Morphol.* **234**, 197–212 (1997).

47. S. M. Reilly, J. S. Willey, A. R. Biknevicius, R. W. Blob, Hindlimb function in the alligator: Integrating movements, motor patterns, ground reaction forces and bone strain of terrestrial locomotion. *J. Exp. Biol.* **208**, 993–1009 (2005).

48. J. L. Hicks, T. K. Uchida, A. Seth, A. Rajagopal, S. L. Delp, Is my model good enough? Best practices for verification and validation of musculoskeletal models and simulations of movement. *J. Biomech. Eng.* **137**, 020905 (2015).

49. V. Allen, R. M. Elsey, N. Jones, J. Wright, J. R. Hutchinson, Functional specialization and ontogenetic scaling of limb anatomy in *Alligator mississippiensis*. *J. Anat.* **216**, 423–445 (2010).

50. V. Allen, J. Molnar, W. Parker, A. Pollard, G. Nolan, J. R. Hutchinson, Comparative architectural properties of limb muscles in Crocodylidae and Alligatoridae and their relevance to divergent use of asymmetrical gaits in extant Crocodylia. *J. Anat.* **225**, 569–582 (2015).

51. W. Herzog, T. R. Leonard, Validation of optimization models that estimate the forces exerted by synergistic muscles. *J. Biomech.* **24**, 31–39 (1991).

52. A. Kian, C. Pizzolato, M. Halaki, K. Ginn, D. Lloyd, D. Reed, D. Ackland, Static optimization underestimates antagonist muscle activity at the glenohumeral joint: A musculoskeletal modeling study. *J. Biomech.* **97**, 109348 (2019).

53. P. Dodson, Functional and ecological significance of relative growth in *Alligator*. *J. Zool.* **175**, 315–355 (1975).

54. H. R. Bustard, L. A. K. Singh, Studies on the Indian gharial *Gavialis gangeticus* (Gmelin) (Reptilia, Crocodilia) change in terrestrial locomotory pattern with age. *J. Bombay Nat. Hist. Soc.* **74**, 534–537 (1977).

55. J. R. Hutchinson, D. Felkler, K. Houston, Y.-M. Chang, J. Brueggen, D. Kledzik, K. A. Vliet, Divergent evolution of terrestrial locomotor abilities in extant Crocodylia. *Sci. Rep.* **9**, 19302 (2019).

56. D. Suzuki, K. Chiba, Y. Tanaka, S. Hayashi, Myology of crocodiles III: Pelvic girdle and hindlimb. *Fossils* **90**, 37–60 (2011).

57. R. Wilhite, "A detailed anatomical study of m. caudofemoralis longus in *Alligator mississippiensis*," in *Ruling Reptiles: Crocodylian Biology and Archosaur Paleobiology* (Indiana Univ. Press, 2023), pp. 80–99.

58. K. Ito, T. Kinugasa, K. Chiba, Y. Okuda, R. Takasaki, S. Hida, T. Okoshi, R. Hayashi, K. Yoshida, K. Osuka, The robotic approach to the passive interlocking mechanism in the hindlimb musculoskeletal system of *Crocodylus porosus*. *Adv. Robot.* **37**, 1187–1197 (2023).

59. L. Sherwood, H. Klandorf, P. H. Yancey, *Animal Physiology: From Genes to Organisms* (Brooks/Cole, 2013).

60. J. P. Charles, O. Cappellari, J. R. Hutchinson, A dynamic simulation of musculoskeletal function in the mouse hindlimb during trotting locomotion. *Front. Bioeng. Biotechnol.* **6**, 61 (2018).

61. R. G. Ellis, J. W. Rankin, J. R. Hutchinson, Limb kinematics, kinetics and muscle dynamics during the sit-to-stand transition in greyhounds. *Front. Bioeng. Biotechnol.* **6**, 162 (2018).

62. J. R. Hutchinson, Biomechanical modeling and sensitivity analysis of bipedal running ability. I. Extant taxa. *J. Morphol.* **262**, 421–440 (2004).

63. Y. Lin, J. W. Rankin, L. P. Lamas, M. Moazen, J. R. Hutchinson, Hindlimb kinematics, kinetics and muscle dynamics during sit-to-stand and sit-to-walk transitions in emus (*Dromaius novaehollandiae*). *J. Exp. Biol.* **227**, jeb247519 (2024).

64. J. W. Rankin, J. Rubenson, J. R. Hutchinson, Inferring muscle functional roles of the ostrich pelvic limb during walking and running using computer optimization. *J. R. Soc. Interface* **13**, 20160035 (2016).

65. K. Steudel, The work and energetic cost of locomotion: I. The effects of limb mass distribution in quadrupeds. *J. Exp. Biol.* **154**, 273–285 (1990).

66. B. M. Kilbourne, L. C. Hoffman, Energetic benefits and adaptations in mammalian limbs: Scale effects and selective pressures. *Evolution* **69**, 1546–1559 (2015).

67. J. O. Farlow, G. R. Hurlburt, R. M. Elsey, A. R. C. Britton, W. Langston Jr., Femoral dimensions and body size of *Alligator mississippiensis*: Estimating the size of extinct mesoeucrocodylians. *J. Vertebr. Paleontol.* **25**, 354–369 (2005).

68. J. D. Currey, The evolution of the mechanical properties of amniote bone. *J. Biomech.* **20**, 1035–1044 (1987).

69. C. Basu, J. R. Hutchinson, Low effective mechanical advantage of giraffes' limbs during walking reveals trade-off between limb length and locomotor performance. *Proc. Natl. Acad. Sci. U.S.A.* **119**, e2108471119 (2022).

70. T. J. M. Dick, C. J. Clemente, Where have all the giants gone? How animals deal with the problem of size. *PLOS Biol.* **15**, e2000473 (2017).

71. J. R. Hutchinson, The evolutionary biomechanics of locomotor function in giant land animals. *J. Exp. Biol.* **224**, jeb217463 (2021).

72. L. Ren, C. E. Miller, R. Lair, J. R. Hutchinson, Integration of biomechanical compliance, leverage, and power in elephant limbs. *Proc. Natl. Acad. Sci. U.S.A.* **107**, 7078–7082 (2010).

73. M. Zwafing, S. Lautenschlager, O. E. Demuth, J. A. Nyakatura, Modeling sprawling locomotion of the stem amniote *Orobates*: An examination of hindlimb muscle strains and validation using extant *Caiman*. *Front. Ecol. Evol.* **9**, 659039 (2021).

74. D. R. Schwimmer, *King of the Crocodylians* (Indiana Univ. Press, 2002).

75. A. P. Cossette, C. A. Brochu, A systematic review of the giant alligatoroid *Deinosuchus* from the Campanian of North America and its implications for the relationships at the root of Crocodylia. *J. Vertebr. Paleontol.* **40**, e1767638 (2020).

76. J. D. Walter, T. Massonne, A. L. S. Paiva, J. E. Martin, M. Delfino, M. Rabi, Expanded phylogeny elucidates *Deinosuchus* relationships, crocodylian osmoregulation and body-size evolution. *Commun. Biol.* **8**, 611 (2025).

77. D. R. Schwimmer, "Bite marks of the giant crocodylian *Deinosuchus* on Late Cretaceous (Campanian) bones," in *Crocodyle Tracks and Traces*, vol. 51 of *New Mexico Museum of Natural History and Science Bulletins* (New Mexico Museum of Natural History and Science, 2010), pp. 183–190.

78. H. E. Rivera-Sylva, E. Frey, J. R. Guzmán-Gutiérrez, Evidence of predation on the vertebra of a hadrosaurid dinosaur from the Upper Cretaceous (Campanian) of Coahuila, Mexico. *Carnets de Géologie* **9**, 1–6 (2009).

79. J. S. Willey, A. R. Biknevicius, S. M. Reilly, K. D. Earls, The tale of the tail: Limb function and locomotor mechanics in *Alligator mississippiensis*. *J. Exp. Biol.* **207**, 553–563 (2004).

80. J. A. Nyakatura, E. Andrada, S. Curth, M. S. Fischer, Bridging "Romer's gap": Limb mechanics of an extant belly-dragging lizard inform debate on tetrapod locomotion during the Early Carboniferous. *Evol. Biol.* **41**, 175–190 (2014).

81. F. E. Zajac, R. R. Neptune, S. A. Kautz, Biomechanics and muscle coordination of human walking: Part I: Introduction to concepts, power transfer, dynamics and simulations. *Gait Posture* **16**, 215–232 (2002).

82. K. M. Steele, M. S. DeMers, M. H. Schwartz, S. L. Delp, Compressive tibiofemoral force during crouch gait. *Gait Posture* **35**, 556–560 (2012).

83. M. T. Carrano, Locomotion in non-avian dinosaurs: Integrating data from hindlimb kinematics, in vivo strains, and bone morphology. *Paleobiology* **24**, 450–469 (1998).

84. M. T. Butcher, B. J. White, N. B. Hudzik, W. C. Gosnell, J. H. A. Parrish, R. W. Blob, *In vivo* strains in the femur of the Virginia opossum (*Didelphis virginiana*) during terrestrial locomotion: Testing hypotheses of evolutionary shifts in mammalian bone loading and design. *J. Exp. Biol.* **214**, 2631–2640 (2011).

85. R. W. Blob, A. A. Biewener, Mechanics of limb bone loading during terrestrial locomotion in the green iguana (*Iguana iguana*) and American alligator (*Alligator mississippiensis*). *J. Exp. Biol.* **204**, 1099–1122 (2001).

86. D. W. E. Hone, M. J. Benton, The evolution of large size: How does Cope's Rule work? *Trends Ecol. Evol.* **20**, 4–6 (2005).

87. P. M. Sander, A. Christian, M. Clauss, R. Fechner, C. T. Gee, E.-M. Griebeler, H.-C. Gunga, J. Hummel, H. Mallison, S. F. Perry, H. Preuschoft, O. W. M. Rauhut, K. Remes, T. Tütken, O. Wings, U. Witzel, Biology of the sauropod dinosaurs: The evolution of gigantism. *Biol. Rev.* **86**, 117–155 (2011).

88. R. B. Sookias, R. J. Butler, R. B. J. Benson, Rise of dinosaurs reveals major body-size transitions are driven by passive processes of trait evolution. *Proc. R. Soc. B Biol. Sci.* **279**, 2180–2187 (2012).

89. J. Benoit, A. J. Midzuk, Estimating the endocranial volume and body mass of *Anteosaurus*, *Jonkeria*, and *Moschops* (Dinocephalia, Therapsida) using 3D sculpting. *Palaeontol. Electron.* **27**, 1–11 (2024).

90. M. Romano, B. Rubidge, First 3D reconstruction and volumetric body mass estimate of the tapinocephalid dinocephalian *Tapinocaninus pamelae* (Synapsida: Therapsida). *Hist. Biol.* **33**, 498–505 (2021).

91. M. Romano, F. Manucci, B. Rubidge, M. J. Van den Brandt, Volumetric body mass estimate and *in vivo* reconstruction of the Russian pareiasaur *Scutosaurus karpinskii*. *Front. Ecol. Evol.* **9**, 692035 (2021).

92. B. S. Rubidge, R. Govender, M. Romano, The postcranial skeleton of the basal tapinocephalid dinocephalian *Tapinocaninus pamelae* (Synapsida: Therapsida) from the South African Karoo Supergroup. *J. Syst. Palaeontol.* **17**, 1767–1789 (2019).

93. M. J. Van den Brandt, M. O. Day, F. Manucci, P. A. Viglietti, K. D. Angielczyk, M. Romano, First volumetric body mass estimate and a new in vivo 3D reconstruction of the oldest Karoo pareiasaur *Bradysaurus baini*, and body size evolution in Pareiasauria. *Hist. Biol.* **36**, 587–601 (2024).

94. M. A. Wright, T. J. Cavanaugh, S. E. Pierce, Volumetric versus element-scaling mass estimation and its application to Permo-Triassic tetrapods. *Integr. Org. Biol.* **6**, obae034 (2024).

95. C. Apaldetti, R. N. Martínez, I. A. Cerda, D. Pol, O. Alcober, An early trend towards gigantism in Triassic sauropodomorph dinosaurs. *Nat. Ecol. Evol.* **2**, 1227–1232 (2018).

96. T. Sulej, G. Niedźwiedzki, An elephant-sized Late Triassic synapsid with erect limbs. *Science* **363**, 78–80 (2019).

97. J. L. Carballido, D. Pol, A. Otero, I. A. Cerda, L. Salgado, A. C. Garrido, J. Ramezani, N. R. Cúneo, J. M. Krause, A new giant titanosaur sheds light on body mass evolution among sauropod dinosaurs. *Proc. R. Soc. B Biol. Sci.* **284**, 20171219 (2017).

98. M. D. D'Emic, The evolution of maximum terrestrial body mass in sauropod dinosaurs. *Curr. Biol.* **33**, R349–R350 (2023).

99. S. M. Gatesy, A. R. Manafzadeh, P. J. Bishop, M. L. Turner, R. E. Kambic, A. R. Cuff, J. R. Hutchinson, A proposed standard for quantifying 3-D hindlimb joint poses in living and extinct archosaurs. *J. Anat.* **241**, 101–118 (2022).

100. F. E. Zajac, Muscle and tendon: Properties, models, scaling, and application to biomechanics and motor control. *Crit. Rev. Biomed. Eng.* **17**, 359–411 (1989).

101. M. Millard, T. Uchida, A. Seth, S. L. Delp, Flexing computational muscle: Modeling and simulation of musculotendon dynamics. *J. Biomech. Eng.* **135**, 0210051 (2013).

102. A. Seth, J. L. Hicks, T. K. Uchida, A. Habib, C. L. Dembia, J. J. Dunne, C. F. Ong, M. S. DeMers, A. Rajagopal, M. Millard, S. R. Hamner, E. M. Arnold, J. R. Yong, S. K. Lakshmikanth, M. A. Sherman, J. P. Ku, S. L. Delp, OpenSim: Simulating musculoskeletal dynamics and neuromuscular control to study human and animal movement. *PLOS Comput. Biol.* **14**, e1006223 (2018).

103. S. Medler, Comparative trends in shortening velocity and force production in skeletal muscles. *Am. J. Physiol. Regul. Integr. Comp. Physiol.* **283**, R368–R378 (2002).

104. K. B. Michel, T. G. West, M. A. Daley, V. R. Allen, J. R. Hutchinson, Appendicular muscle physiology and biomechanics in *Crocodylus niloticus*. *Integr. Org. Biol.* **2**, obaa038 (2020).

105. F. E. Nelson, A. M. Gabaldón, T. J. Roberts, Force–velocity properties of two avian hindlimb muscles. *Comp. Biochem. Physiol. A Mol. Integr. Physiol.* **137**, 711–721 (2004).

106. J. Méndez, A. Keys, Density and composition of mammalian muscle. *Metabolism* **9**, 184–188 (1960).

107. K. Manal, T. S. Buchanan, Subject-specific estimates of tendon slack length: A numerical method. *J. Appl. Biomech.* **20**, 195–203 (2004).

108. P. J. Bishop, K. B. Michel, A. Falisse, A. R. Cuff, V. R. Allen, F. D. Groote, J. R. Hutchinson, Computational modelling of muscle fibre operating ranges in the hindlimb of a small ground bird (*Eudromia elegans*), with implications for modelling locomotion in extinct species. *PLOS Comput. Biol.* **17**, e1008843 (2021).

109. A. M. Heers, J. W. Rankin, J. R. Hutchinson, Building a bird: Musculoskeletal modeling and simulation of wing-assisted incline running during avian ontogeny. *Front. Bioeng. Biotechnol.* **6**, 140 (2018).

110. M. Iijima, T. Kubo, Allometric growth of limb and body proportions in crocodylians. *J. Zool.* **309**, 200–211 (2019).

111. W. I. Sellers, S. B. Pond, C. A. Brassey, P. L. Manning, K. T. Bates, Investigating the running abilities of *Tyrannosaurus rex* using stress-constrained multibody dynamic analysis. *PeerJ* **5**, e3420 (2017).

112. V. R. Allen, R. E. Kambic, S. M. Gatesy, J. R. Hutchinson, Gearing effects of the patella (knee extensor muscle sesamoid) of the helmeted guineafowl during terrestrial locomotion. *J. Zool.* **303**, 178–187 (2017).

113. O. E. Demuth, A. L. A. Wiseman, J. van Beesel, H. Mallison, J. R. Hutchinson, Three-dimensional polygonal muscle modelling and line of action estimation in living and extinct taxa. *Sci. Rep.* **12**, 3358 (2022).

114. W. D. Pilkey, *Analysis and Design of Elastic Beams: Computational Methods* (John Wiley & Sons, 2002).

115. M. Iijima, T. Kubo, Vertebrae-based body length estimation in crocodylians and its implication for sexual maturity and the maximum sizes. *Integr. Org. Biol.* **2**, obaa042 (2020).

116. P. L. Knüsel, "Beiträge zur morphologie und funktion der crocodiliden-extremitäten," Inaugural dissertation, University of Freiberg, Switzerland (1944). [Studies on the morphology and function of crocodilian limbs]

117. V. J. Livingston, M. F. Bonnan, R. M. Elsey, J. L. Sandrik, D. R. Wilhite, Differential limb scaling in the American alligator (*Alligator mississippiensis*) and its implications for archosaur locomotor evolution. *Anat. Rec.* **292**, 787–797 (2009).

118. H. Wickham, *ggplot2: Elegant Graphics for Data Analysis* (Springer-Verlag, 2016).

119. H. Wickham, M. Averick, J. Bryan, W. Chang, L. D. McGowan, R. François, G. Grolemund, A. Hayes, L. Henry, J. Hester, M. Kuhn, T. L. Pedersen, E. Miller, S. M. Bache, K. Müller,

J. Ooms, D. Robinson, D. P. Seidel, V. Spinu, K. Takahashi, D. Vaughan, C. Wilke, K. Woo, H. Yutani, Welcome to the tidyverse. *J. Open Source Softw.* **4**, 1686 (2019).

120. C. Agostinelli, U. Lund, circular: Circular statistics (2024); https://CRAN.R-project.org/package=circular.

121. D. I. Warton, R. A. Duursma, D. S. Falster, S. Taskinen, smatr 3– an R package for estimation and inference about allometric lines. *Methods Ecol. Evol.* **3**, 257–259 (2012).

122. R Core Team, R: A language and environment for statistical computing, R Foundation for Statistical Computing (2022); www.R-project.org/.

123. A. A. Biewener, Musculoskeletal design in relation to body size. *J. Biomech.* **24**, 19–29 (1991).

124. A. A. Biewener, Locomotory stresses in the limb bones of two small mammals: The ground squirrel and chipmunk. *J. Exp. Biol.* **103**, 131–154 (1983).

125. A. A. Biewener, R. Blickhan, Kangaroo rat locomotion: Design for elastic energy storage or acceleration ? *J. Exp. Biol.* **140**, 243–255 (1988).

126. C. T. Rubin, L. E. Lanyon, Dynamic strain similarity in vertebrates; an alternative to allometric limb bone scaling. *J. Theor. Biol.* **107**, 321–327 (1984).

127. R. McN. Alexander, A. Vernon, The mechanics of hopping by kangaroos (Macropodidae). *J. Zool.* **177**, 265–303 (1975).

128. A. A. Biewener, C. R. Taylor, Bone strain: A determinant of gait and speed? *J. Exp. Biol.* **123**, 383–400 (1986).

129. C. T. Rubin, L. E. Lanyon, Limb mechanics as a function of speed and gait: A study of functional strains in the radius and tibia of horse and dog. *J. Exp. Biol.* **101**, 187–211 (1982).

130. R. McN. Alexander, The mechanics of jumping by a dog (*Canis familiaris*). *J. Zool.* **173**, 549–573 (1974).

131. A. A. Biewener, J. Thomason, A. Goodship, L. E. Lanyon, Bone stress in the horse forelimb during locomotion at different gaits: A comparison of two experimental methods. *J. Biomech.* **16**, 565–576 (1983).

132. A. A. Biewener, J. J. Thomason, L. E. Lanyon, Mechanics of locomotion and jumping in the horse (*Equus*): *In vivo* stress in the tibia and metatarsus. *J. Zool.* **214**, 547–565 (1988).

133. R. McN. Alexander, G. M. O. Maloiy, B. Hunter, A. S. Jayes, J. Nturibi, Mechanical stresses in fast locomotion of buffalo (*Syncews coffer*) and elephant (*Loxodonta africana*). *J. Zool.* **189**, 135–144 (1979).

134. A. R. Manafzadeh, S. M. Gatesy, Paleobiological reconstructions of articular function require all six degrees of freedom. *J. Anat.* **239**, 1516–1524 (2021).

135. E. L. Brainerd, D. B. Baier, S. M. Gatesy, T. L. Hedrick, K. A. Metzger, S. L. Gilbert, J. J. Crisco, X-ray reconstruction of moving morphology (XROMM): Precision, accuracy and applications in comparative biomechanics research. *J. Exp. Zool. A Ecol. Genet. Physiol.* **313A**, 262–279 (2010).

136. S. M. Gatesy, D. B. Baier, F. A. Jenkins, K. P. Dial, Scientific rotoscoping: A morphology-based method of 3-D motion analysis and visualization. *J. Exp. Zool. A Ecol. Genet. Physiol.* **313A**, 244–261 (2010).

137. S. Hattori, T. Tsuihiji, Homology and osteological correlates of pedal muscles among extant sauropsids. *J. Anat.* **238**, 365–399 (2021).

138. L. Cong, L. Hou, X. C. Wu, J. F. Hou, *The Gross Anatomy of Alligator Sinensis Fauvel* (Science Press, 1998).

139. A. S. Romer, Crocodilian pelvic muscles and their avian and reptilian homologues. *Bull. Am. Mus. Nat. Hist.* **48**, 533–552 (1923).

140. P. J. Bishop, A. R. Cuff, J. R. Hutchinson, How to build a dinosaur: Musculoskeletal modeling and simulation of locomotor biomechanics in extinct animals. *Paleobiology* **47**, 1–38 (2021).
